# A systematic scoping review of approaches to teaching and assessing empathy in medicine

**DOI:** 10.1186/s12909-021-02697-6

**Published:** 2021-05-22

**Authors:** Yi Cheng Zhou, Shien Ru Tan, Chester Guan Hao Tan, Matthew Song Peng Ng, Kia Hui Lim, Lorraine Hui En Tan, Yun Ting Ong, Clarissa Wei Shuen Cheong, Annelissa Mien Chew Chin, Min Chiam, Elisha Wan Ying Chia, Crystal Lim, Limin Wijaya, Anupama Roy Chowdhury, Jin Wei Kwek, Warren Fong, Nagavalli Somasundaram, Eng Koon Ong, Stephen Mason, Lalit Kumar Radha Krishna

**Affiliations:** 1 Yong Loo Lin School of Medicine, National University of Singapore, NUHS Tower Block, 1E Kent Ridge Road, Level 11, Singapore, 119228 Singapore; 2 Division of Supportive and Palliative Care, National Cancer Centre Singapore, 11 Hospital Cr, Singapore, 169610 Singapore; 3 Medical Library, National University of Singapore Libraries, National University of Singapore Blk MD6, Centre for Translational Medicine, 14 Medical Dr, #05-01, Singapore, 117599 Singapore; 4 Division of Cancer Education, National Cancer Centre Singapore, 11 Hospital Cr, Singapore, 169610 Singapore; 5 Duke-NUS Medical School, National University of Singapore, 8 College Rd, Singapore, 169857 Singapore; 6 Medical Social Services, Singapore General Hospital, 16 College Road, Block 3 Level 1, Singapore, 169854 Singapore; 7 Department of Infectious Diseases, Singapore General Hospital, 16 College Road, Block 6 Level 7, Singapore, 169854 Singapore; 8 Department of General Medicine, Sengkang General Hospital, 110 Sengkang East Way, Singapore, 544886 Singapore; 9 Division of Oncologic Imaging, National Cancer Centre Singapore, 11 Hospital Cr, Singapore, 169610 Singapore; 10 Department of Rheumatology and Immunology, Singapore General Hospital, 16 College Road, Block 6 Level 9, Singapore, 169854 Singapore; 11 Division of Medical Oncology, National Cancer Centre Singapore, 11 Hospital Cr, Singapore, 169610 Singapore; 12 Palliative Care Institute Liverpool, Academic Palliative & End of Life Care Centre, University of Liverpool, Cancer Research Centre, University of Liverpool, 200 London Rd, Liverpool, L3 9TA UK; 13 Centre of Biomedical Ethics, National University of Singapore, Blk MD 11, 10 Medical Drive, #02-03, Singapore, 117597 Singapore; 14 PalC, The Palliative Care Centre for Excellence in Research and Education, PalC c/o Dover Park Hospice, 10 Jalan Tan Tock Seng, Singapore, 308436 Singapore

**Keywords:** Empathy, Nurturing empathy, Medical schools, Medical education

## Abstract

**Background:**

Empathy is pivotal to effective clinical care. Yet, the art of nurturing and assessing empathy in medical schools is rarely consistent and poorly studied. To inform future design of programs aimed at nurturing empathy in medical students and doctors, a review is proposed.

**Methods:**

This systematic scoping review (SSR) employs a novel approach called the Systematic Evidence Based Approach (SEBA) to enhance the reproducibility and transparency of the process. This 6-stage SSR in SEBA involved three teams of independent researchers who reviewed eight bibliographic and grey literature databases and performed concurrent thematic and content analysis to evaluate the data.

**Results:**

In total, 24429 abstracts were identified, 1188 reviewed, and 136 included for analysis. Thematic and content analysis revealed five similar themes/categories. These comprised the 1) definition of empathy, 2) approaches to nurturing empathy, 3) methods to assessing empathy, 4) outcome measures, and 5) enablers/barriers to a successful curriculum.

**Conclusions:**

Nurturing empathy in medicine occurs in stages, thus underlining the need for it to be integrated into a formal program built around a spiralled curriculum. We forward a framework built upon these stages and focus attention on effective assessments at each stage of the program. Tellingly, there is also a clear need to consider the link between nurturing empathy and one’s professional identity formation. This foregrounds the need for more effective tools to assess empathy and to better understand their role in longitudinal and portfolio based learning programs.

**Supplementary Information:**

The online version contains supplementary material available at 10.1186/s12909-021-02697-6.

## Background

A physician’s ability to demonstrate empathy strengthens doctor-patient relationships [1, 2], boosts patient outcomes [3, 4], patient satisfaction [2, 5], increases professional satisfaction [6, 7], improves clinical competence [8, 9] and reduces potential burnout [10, 11].

Yet, despite these benefits and evidence of diminishing empathy midway through medical school [12, 13], empathy remains poorly nurtured in medical school and postgraduation [9, 14–19]. These gaps have been attributed to the lack of an accepted definition of empathy that fully considers cognitive, affective and behavioural components highlighted in current literature [20]. Inconsistencies in the structuring of programs aimed at nurturing empathy and the lack of effective assessment methods further exacerbate the issue [14, 18].

To enhance understanding of how empathy may best nurtured and to address prevailing knowledge gaps, we propose a review of prevailing efforts to nurture and assess empathy amongst physicians and medical students.

## Methodology

The reflexive nature of systematic scoping reviews (SSR)s and their lack of structure raises concerns over their reproducibility and transparency. To overcome this, we adopted Krishna’s novel Systematic Evidence Based Approach (SEBA) [21–23]. Compared to other existing SSR approaches [24], SEBA acknowledges the complex nature of empathy and the need to evaluate how empathy is nurtured and assessed in different programs, involving different education and healthcare structures and funding. SEBA’s constructivist approach and relativist lens allow for a multi-dimensional, transparent, and reproducible method of studying empathy – a personalised, socioculturally and contextually informed concept. This SSR in SEBA also facilitates systematic extraction, synthesis and summary of actionable and applicable information across a diverse range of study formats and overcomes a paucity of articles on this subject.

**Fig. 1:**
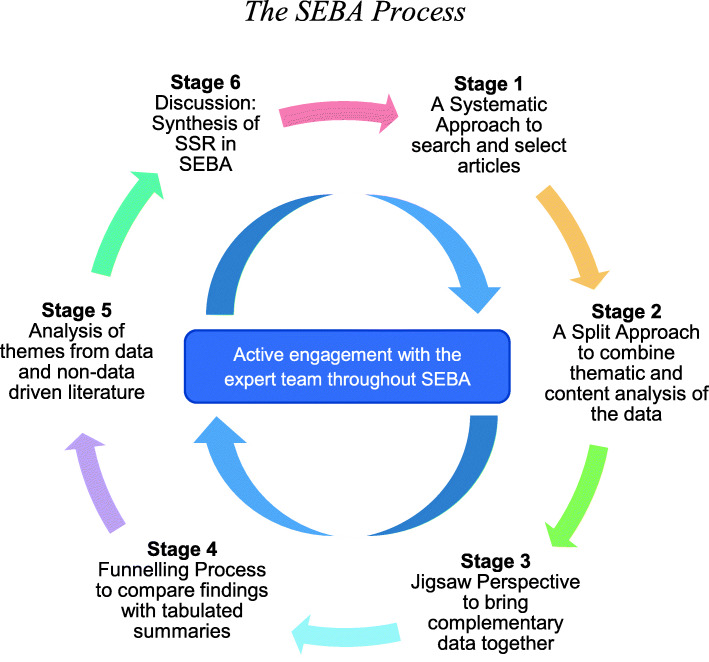
The SEBA Process

To enhance accountability within the SEBA methodology, the research process is overseen by a team of experts comprising of a medical librarian from the National University of Singapore’s (NUS) Yong Loo Lin School of Medicine (YLLSoM), educational, clinical and research experts from the National Cancer Centre Singapore (NCCS), the Palliative Care Institute Liverpool, YLLSoM and Duke-NUS Medical School (henceforth the expert team). The SEBA process consists of the following six stages: 1) Systematic Approach, 2) Split Approach, 3) Jigsaw Perspective, 4) Funnelling Process 5) Analysis of data and non-data driven literature, and 6) Discussion. This is outlined in Fig. 1 and will be further elaborated.

### Stage 1 of SEBA: Systematic approach 

#### Determining title and research question

Guided by the expert team, the research team determined the primary research question to be “*How effective are current methods to nurture empathy in doctors and medical students?*” and the secondary research question to be “*what are the features of these programs*?”. These questions were designed on the Population, Concept, and Context (PCC) elements of the inclusion criteria [25] and were concurrently guided by the PRISMA-P 2015 checklist [26].

#### Inclusion and exclusion criteria

The PICOS format was used to guide the research process, as outlined in Table 1.

**Table 1:** PICOS, Inclusion Criteria and Exclusion Criteria Applied to Database Search

	Inclusion	Exclusion
**Population**	• Doctors/Physicians • Medical Students	• Allied health specialties such as Pharmacy, Dietetics, Chiropractic, Midwifery, Podiatry, Speech Therapy, Occupational and Physiotherapy • Non-medical specialties such as Clinical and Translational Science, Alternative and Traditional Medicine, Veterinary, Dentistry
**Intervention**	• Interventions (with specific outcomes i.e. qualitative/quantitative)	• Sympathy • Self-compassion • Compassion • If empathy is only minor component of scale/assessment (eg, surveys) • Transference/Countertransference • Psychotherapy
**Comparison**	NA	NA
**Outcomes**	• Impact of curricula on participants, patients, or host organisation	NA
**Study Design**	• All study designs and article types were included: - Mixed methods research, meta-analyses, systematic reviews, randomized controlled trials, cohort studies, case-control studies, cross-sectional studies, and descriptive papers - Grey Literature / electronic and print information not controlled by commercial publishing - Case reports and series, ideas, editorials, conference abstracts, and perspectives	• Non-English articles without English translations

#### Searching

To enhance trustworthiness of this approach, five members of the research team carried out independent searches between 14th February and 24th April 2020 for articles in PubMed, Embase, PsychInfo, CINAHL, Scopus, Cochrane, OpenGrey and ProQuest Dissertations using identical inclusion and exclusion criteria and search terms. The PubMed search strategy may be found in Supplementary file 1. All articles published up to 31st December 2019 were included. The results of these independent searches were discussed online and consensus was achieved on the final list of articles to be included using Sandelowski and Barroso [27]’s ‘negotiated consensual validation’ approach.

Additional articles that meet the PICOS requirement were obtained by ancestry searching/ forward tracing of the references in the first set of included articles.

#### PRISMA

**Fig. 2:**
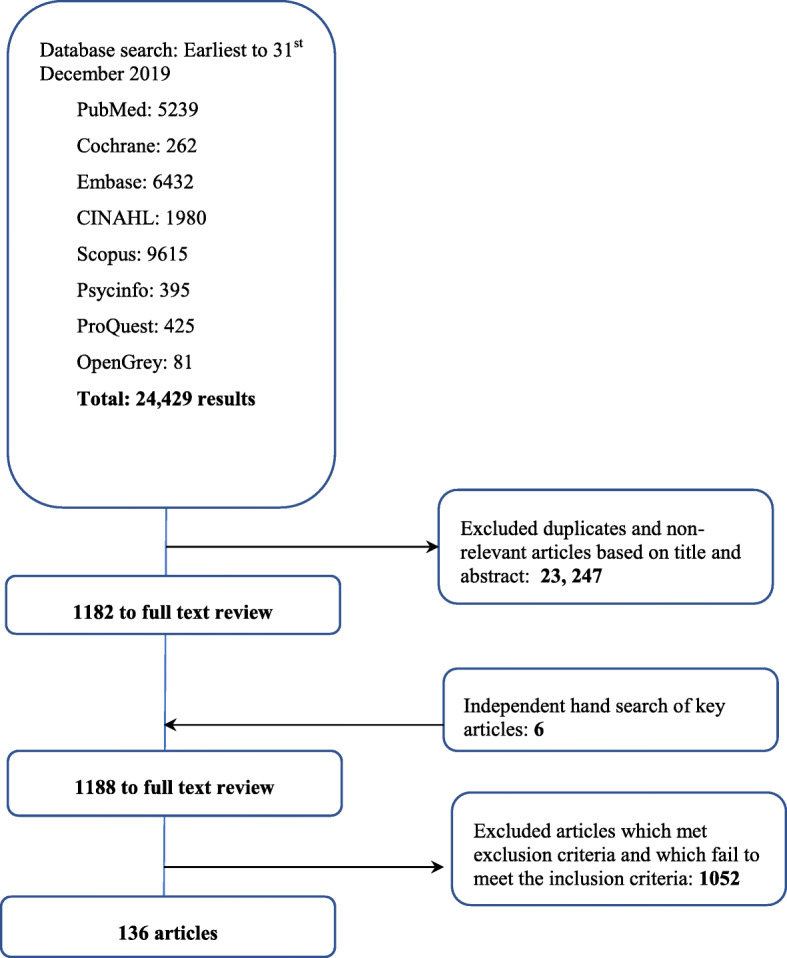
PRISMA Flowchart

The research team identified 24,429 abstracts from the eight databases, 1188 articles were reviewed, and 136 articles were included (Fig. 2).

### Stage 2 of SEBA: Split approach

Krishna’s Split Approach was employed to enhance the trustworthiness of the data analyses [21, 28]. The Split Approach saw two independent teams of at least three experienced researchers carrying out concurrent analysis of the included articles using Braun and Clarke [29]’s approach to thematic analysis and Hsieh and Shannon [30]’s approach to directed content analysis. Use of the Split Approach was employed in acknowledgment that a combination of these approaches reduces the omission of new findings and minimises the neglect of negative findings.

The categories for Hsieh and Shannon [30]’s approach to directed content analysis was drawn from Batt-Rawden, Chisolm [20] “*Teaching Empathy to Medical Students: An Updated, Systematic Review*”. Deductive category application was used to determine if any data was not captured by the pre-determined categories [31].

### Stage 3 of SEBA: Jigsaw perspective

To present a holistic perspective of methods to nurture empathy, the Jigsaw Perspective pieces the themes identified through use of thematic analysis and categories used in directed content analysis in order to facilitate their effective interpretation and analysis.

### Stage 4 of SEBA: Funnelling process

All 136 included articles were then independently reviewed and summarised using Wong et al.’s *“RAMESES Publication Standards: Meta-narrative Reviews”* [32] and Popay et al.’s *“Guidance on the conduct of narrative synthesis in systematic reviews”* [33] These tabulated summaries ensure that key discussion points and contradictory views within the included articles are not lost (Supplementary file 2).

The themes/categories identified through the Split Approach were then compared with the tabulated summaries to prevent the loss of contradictory data and also served as a form of data triangulation. The verified themes/categories which will be presented in the Results section also formed the basis of the narrative synthesised in the Discussion section.

### Stage 5 of SEBA: Analysis of data and non-data driven literature

In keeping with SEBA’s iterative process and active engagement with the expert team, the findings were discussed with the expert team and concerns were raised over the influence of grey literature on the results as these were neither peer reviewed nor clearly evidence based. Therefore, the research team differentiated grey literature such as correspondence, letters, editorials and perspective pieces extracted from academic databases, from data-driven and research-based peer reviewed articles. Both were analysed independently, and the themes derived from the grey literature were found to be in agreement with themes from the peer-reviewed literature [21–23, 34–37].

## Results

The research team identified 24,429 abstracts were identified from eight databases, 1188 articles were reviewed and 136 articles were included in this review as shown in Fig. 2.

As the final five themes/categories identified through the Split Approach, Jigsaw Perspective and Funnelling Process were determined to be parallel in nature, they will be discussed in tandem for ease of understanding. The five themes/categories identified were the 1) definition of empathy, 2) approaches to nurturing empathy, 3) methods to assessing empathy, 4) outcome measures, and 5) enablers/barriers to a successful curriculum.

### Definition of empathy

Overall, 35 articles stated that empathy was poorly defined in the literature [3, 6, 7, 10, 16, 17, 38–66]. Yet, analysis of prevailing accounts allow discernment of common characteristics amongst current accounts of empathy. Thus in the absence of a widely accepted definition of empathy, its cognitive, affective, behavioural, intrinsic and self-regulatory components must be considered.

The cognitive component suggests that empathy is “standing in the patient’s shoes” without confusing the patient’s experience as one’s own [67]. It hinges on identifying and understanding the patient’s perspective and mental state without losing objectivity. Sixty eight articles adopted variations of this approach [1, 2, 4, 5, 9, 11, 12, 14, 15, 17–20, 48, 60, 68–120].

The affective component sees recognition of the patient’s emotions and the offering of a suitable response [1, 2, 9, 11, 12, 15, 18, 20, 48, 67, 70, 79, 83, 88, 91–94, 98, 102, 104, 105, 108, 110–114, 118, 120–124]. The affective component is closely related to behavioural components of empathy which entail verbal and non-verbal communication of another person’s inner state [2, 11–13, 15, 18, 48, 60, 70, 71, 73, 76, 78, 83, 93, 95, 102, 106–111, 113, 115, 120, 125–128] and their intrinsic motivation to help others to reduce their distress [2, 4, 11, 12, 60, 83, 93, 106, 108, 113–115]. Decety and Meyer [129] and Airagnes et al. [67] argue that responding to the emotions and needs of others underscores the presence of self-regulation [129] and the ability to avoid “confusion between self and others” [67].

### Approaches used

This theme/category revolves around the benefits of nurturing empathy in medicine for the patient and the physician, the methods of realising these benefits and the contents of these programs (Table 2).

**Table 2:** Benefits of Greater Physician Empathy for the Physician and Patient

Benefits to	Elaboration	References
**Patients**	Better understanding of the patient	[10, 11, 14, 42, 58, 63, 69, 78, 86, 100, 101, 103, 130]
	Improved patient satisfaction	[1–7, 9, 10, 12, 15, 20, 38, 39, 45, 50, 53, 68–72, 76, 78, 80, 83, 87, 88, 93, 94, 96, 104, 108, 112, 114, 115, 118, 121, 124, 126, 127, 131–137]
	Greater adherence to therapy	[1, 5, 6, 9, 10, 12, 14, 20, 38, 39, 43–45, 47, 50, 53, 68, 70–72, 75, 76, 78, 80, 81, 83, 87, 91, 93, 94, 96, 104, 105, 108, 112–115, 121, 124, 125, 127, 131, 132, 137, 138]
	Better clinical outcomes	[1–10, 14–16, 19, 20, 39, 41, 43–46, 53, 58, 64–70, 72, 76, 78–80, 88, 93, 94, 96, 104, 105, 108–114, 117–121, 123–125, 127, 128, 131, 137, 139–141]
	Patient empowerment	[69, 128]
**Physicians**	Lower malpractice liability	[4, 9, 10, 12, 15, 20, 45, 71, 75, 76, 78, 93, 105, 108, 112, 113, 118, 126, 131, 132]
	Improved well-being of physicians	[1–3, 5–7, 9, 10, 12, 14, 17, 50, 52, 61, 64, 68, 70, 74, 76, 78, 83, 84, 94, 105, 108, 109, 113, 114, 118]
	Fosters a better physician-patient relationship	[2, 4, 10, 19, 50, 56, 58, 60, 65, 66, 78, 80, 105, 107, 108, 111, 114, 115, 118, 119, 121, 125, 126, 138, 141]
	Greater clinical competence	[2, 4, 41, 55, 60, 68, 72, 75, 86, 101, 108, 115, 131, 138]

A variety of approaches have been employed to nurture empathy but are not discussed in detail. For ease of reference they are summarised in Table 3.

**Table 3:** Various Teaching Modalities Used to Nurture Empathy in Medical Education

Modalities	References
Didactic teaching sessions	[4, 6, 7, 12, 15, 17, 40, 43, 47, 51, 57, 59, 61, 64, 65, 72, 76, 84, 86, 89, 91, 108, 109, 113, 116, 127–129, 144]
Group discussion	[7, 10, 12, 16, 17, 42, 45, 50, 51, 53, 57–59, 66, 67, 73, 80, 82, 100, 102, 103, 106, 109, 111–113, 116, 128, 142–144]
Role play	[6, 7, 12, 13, 15, 40, 43, 45, 47, 51, 65, 74, 78, 91, 98, 91, 108, 110, 116, 117, 119, 122, 128, 139, 142–144]
Simulated patients	[2, 8, 41, 49, 115, 122, 126, 132, 133, 145]
Simulations and experiential learning	[9, 63, 82, 131, 132, 138, 146, 147, 148]
Virtual patients	[118, 121, 136]
Real patients	[43, 59, 88, 101, 125]
Balint groups	[45, 68, 105, 123, 149, 150]
Multimedia tools	[1, 4, 19, 39, 46, 51, 54, 55, 62, 66, 69, 91, 95, 106, 109, 110, 114, 125, 139]
Arts and humanities	[4, 5, 16, 43, 49, 52, 58, 60, 67, 71–73, 83, 91, 111, 113, 120, 136, 151]
Longitudinal integrated clerkship	[11, 72]

Group discussions on personal experiences [71, 101, 115] and/or simulated scenarios including role play and simulated patients [16, 47, 57, 65] facilitate analysis of empathy [115] and shared experiences [63]. Role play has been found to boost participants’ confidence in communication [44, 118, 142, 144]. The use of the arts and humanities including poetry and literature [49, 57, 83, 136, 139], drawings and paintings [16, 43, 59, 83, 136, 139], reflective writing [49, 83, 136, 139], cultural studies and history [16], film [16], photography [59], and comics [5] have also shown to increase self-awareness and reflection [59].

The topics introduced in the ‘teaching’ of empathy vary significantly. They include mindfulness [17, 43, 78, 95, 105, 112, 115, 127, 133, 140, 148, 151, 152], communication and interpersonal skills [6, 12, 13, 15, 19, 38, 50, 51, 56, 60, 64, 69, 73, 85, 94, 105, 109, 115, 119, 121, 125, 127, 128, 138, 146, 143], and the arts and humanities [4, 5, 16, 43, 49, 57, 59, 72, 83, 139]. Teachings in mindfulness involve meditation and mindful listening [78, 95, 112, 133, 140] whilst communication skills include active listening [73, 125, 128, 138], use of open-ended questions [64], and improving communication among healthcare staff [69]. Arts based curricula include teachings such as principles of art therapy [136], art analysis [112], and social and cultural studies [16].

Critically, empathy was nurtured by facilitating understanding of the concept of empathy [19, 94, 108, 115], underscoring the differences between empathy and sympathy [108, 127], its importance [4, 94, 119] and its role in clinical practice [2, 12, 60, 68, 94, 108, 109].

### Assessment methods used

Assessments of empathy involved self-rated, assessor and/or observer ratings. Whilst the most common assessment tool is the Jefferson Scale of Empathy (JSE), a number of other approaches have also been adopted as highlighted in Table 4.

**Table 4:** Assessment Methods Used to Evaluate Empathy

Type of assessment, and who they were utilised by	Assessment tool	Studies that used the tool
Quantitative self-rated	Jefferson Scale of Empathy	[1, 2, 4, 5, 8–12, 14, 16, 18, 40, 43, 45, 48, 52, 61, 65, 68, 69, 71–73, 75, 80, 81, 87–91, 94, 96, 100, 104, 106, 108, 113, 114, 117, 118, 122, 124, 127, 131–133, 137, 139–141, 142, 148, 149, 152]
	Interpersonal Reactivity Index	[48, 49, 52, 67, 74, 78, 93, 101, 112, 114]
	Balanced Emotional Empathy Scale	[12, 45, 74, 93, 110, 123]
	Toronto Empathy Questionnaire	[18, 47, 49]
	Adapted Empathy Construct Rating Scale	[74, 95, 110]
	Empathy Quotient	[85]
	Groningen Reflection Ability Scale	[78]
	Ekman Facial Decoding Test	[12, 45]
	Social Empathy Index	[82, 84]
	Self-developed Tools	[3, 17, 54, 66, 107, 133, 138, 141, 142, 143, 145]
	Empathic Tendency Scale	[60, 79, 109, 146]
Quantitative assessor- based	Consultation and Relational Empathy measure	[6, 10, 12, 18, 45, 97, 104, 115, 131]
	Jefferson Scale of Patient Perceptions of Physician Empathy	[40, 43, 88, 131]
	Standardised Patient Feedback Form – Part II	[69]
	Quality of Communication through Patient’s Eyes	[6]
	Modified Barret-Lenard Relationship Inventory	[58, 98]
	Self-developed assessment of patient-rated empathy	[38]
Quantitative observer-based	Truax Accurate Empathy Scale	[19, 58, 121]
	Roter’s Interaction Analysis System	[39]
	Empathy Skill Scale	[79]
	Empathy Communication Skill Scale	[60]
	Empathic Communication Coding System	[15, 117, 120]
	Well’s Empathic Communication Test	[116]
	Index of Facilitative Discrimination	[116]
	Modified Scoring Tool used in Theatre Department	[119]
	ComSkill Coding System	[77]
Qualitative observer-based	Interviews	[6, 41, 70, 112]
	Group discussion	[5, 101, 110, 112, 122]
	Evaluation of reflective writing, narratives, or blogs submitted by participants	[9, 43, 51, 55, 63, 66, 75, 84, 103, 133, 142, 153]
	Analysis of artwork	[92, 139]
	Thematic analysis of SP interviews	[13, 42, 50, 64, 77, 99, 119]
	Self-developed questionnaire	[62]
	Qualitative survey feedback	[3, 70]

In some cases, local assessment tools have adapted various elements of established tools such as the social presence questionnaire from the JSE [117], or the “perspective taking” and “empathic concern” subscales of the Interpersonal Reactivity Index (IRI) [49].

### Outcome measures of the curricula

The outcomes of different curricular programs varied. This is in part due to use of diverse approaches, contents, training programs, setting, duration and assessment methods. Table 5 summarises the reported outcomes.

**Table 5:** Qualitative and Quantitative Outcome Measures of the Curricula

**Quantitative outcomes**	**Studies that displayed this outcome**
Quantitative **increase** in overall empathy levels after intervention as measured by the respective tool used	[1–4, 6, 8, 9, 11–13, 15–17, 19, 38–40, 42–45, 47–50, 52–58, 60–65, 67–70, 72–75, 77, 83, 84, 88, 90, 91, 94–96, 98–101, 104–108, 110–125, 130, 133, 134, 137–139, 141, 146, 143–147]
Quantitative **decrease** in empathy levels after intervention	[1, 3, 61, 67, 80, 97, 106, 113, 135, 139]
**No statistically significant change** in empathy levels after intervention	[2, 5, 10–12, 51, 61, 69, 71, 79–81, 84, 85, 89, 97, 104, 106, 109, 110, 112, 127, 128, 132, 133, 135, 142, 148, 149, 151, 152]
**Qualitative outcome**	**Studies**
Participant feedback suggested an improvement in empathy	[102]
Participants understood patient perspectives better	[5, 7, 57, 59]
The intervention helped with professional identity building	[55, 59, 122]
Participants valued empathy more	[5, 105]
Participants were better able to decode facial expression of emotion	[45]
Participants had a greater tendency to see patients’ emotions	[51, 103]
Participants developed better general observational skills	[5, 55]
Patients felt more understood and cared for	[19]

The impact of programs aimed at nurturing empathy are widely reported and vary in their effects. Using the IRI, Sands et al. [101] reported increases in the “perspective taking” and “empathic concern” subscales, Airagnes et al. [67] reported an increase in the “fantasy” subscale, and Winkel et al. [49] reported an increase in “empathic concern”. Smith et al. [113] reported an increase in cognitive empathy using the Questionnaire of Cognitive and Affective Empathy (QCAE), whilst Wellbery et al. [84] reported an increase in mean scores in the “contextual understanding of systemic barriers” domain of the Social Empathy Index (SEI) survey in their respective programs. Using the JSE, Stebbins [69] reported an improvement in the “ability to stand in patient’s shoes” subscale while San-Martín et al. [106], using three different curricula for three populations, concluded that the participants could either have increases in some or all three components of the scale depending on the approach employed [106].

Conversely, Airagnes et al. [67] reported a decrease in the “empathic concern” subscale of the IRI and San-Martín et al. [106] found a decrease in both the “compassionate care” and “walking in patient’s shoes” components of the JSE in the clinical phase of medical school. Bombeke et al. [80] suggest that these reductions in empathy related scores are the result of students witnessing the difference between idealistic teachings in their curriculum and the realities of clinical care and patient interactions. This may in turn have contributed to negative attitudes towards the training they received. Others suggest that a lack of finesse and clarity when using the tools may have contributed to a relative decrease in empathy scores [3, 97, 113, 139]. The apparent gaps in prevailing assessment tools are also highlighted when participants reported no significant change in self-reported scores on the JSE but simulated patients (SPs) and observers rated improved empathy scores [2, 12, 40, 88].

Notably, current tools seem to measure empathy along the different levels of Kirkpatrick’s Hierarchy [154]. This consists of Level 1 (participation), Level 2a (attitudes and perceptions) and Level 2b (knowledge and skills), Level 3 (behavioural change), Level 4a (organisational practice) and Level 4b (patient benefits). Whilst 29 of the 136 articles measured changes at Level 3, 20 focused on Level 4. These are summarised in Table 6.

**Table 6:** Modified Kirkpatrick’s framework (Barr et al.’s six-level classification adaptation) [155]

Kirkpatrick outcome level	Outcome	Studies that achieved this outcome
Level 1	Participant reaction Learners’ views on the learning experience and its interprofessional nature	• Participants reported decreased stress [133, 149, 151] • Participants had a positive experience with the intervention [3, 57, 138]
Level 2a	Change in own attitudes and change in attitudes towards team members of the interprofessional groups	• Increased empathic tendency [130, 135, 146, 150, 153] • Participants reported increased empathy [41, 55, 83] • Improved self-reported ability to show empathy [54, 107]
Level 2b	Change in knowledge or skills Including knowledge and skills related to the interprofessional activity	• Improvement in self-rated empathy scores using validated scales [1, 4, 8, 9, 11, 16, 19, 47–49, 52, 60, 61, 65, 68, 71–73, 75, 78, 79, 82, 87, 90, 94–96, 100, 104, 106, 108, 110, 114, 121, 123, 124, 127, 132, 137, 141] • Increased understanding of empathy from analysis of reflections or artworks [9, 51, 67, 71, 84, 92, 136, 139]
Level 3	Behavioural change Identify individual transfer of interprofessional learning	• Improved empathic communication with standardised patients [2, 5, 7, 13, 15, 17, 40, 46, 50, 56, 60, 64, 88, 91, 99, 111, 112, 115–118, 120, 134, 147]. • Increased confidence with clinical interactions [77, 105, 126, 143, 144]
Level 4a	Change in organisational practice Wider change in organisational practice and delivery of care	• Increased sense of belonging among participants [122] • Reduced participant burnout [59, 140, 152]
Level 4b	Change in clinical outcome Improvement in patient care	• Increased emphatic communication or attitudes with patients [6, 42, 43, 53, 62, 98, 103, 119] • Improved patient satisfaction [38, 39, 85] • Barriers to empathy and administrative changes to curb them were identified by participants [66, 70, 142] • Participants identified lapses in patient care [63] • Improved patient rated empathy score [12]

Twenty one of the 29 studies that focused upon Level 3 of Kirkpatrick’s Hierarchy focused on general communication skills training. Fifteen of these studies employed role play, simulations and/or patient interviews to encourage communication skills [7, 40, 46, 50, 58, 64, 88, 91, 111, 115–117, 120, 125, 143] in a safe practice space [7, 9, 44, 62, 115, 147].

A common feature among the studies aiming at Level 4 of Kirkpatrick’s Hierarchy was that they encouraged participants to consider mindfulness [140, 152] and the patient’s perspective [43, 53, 62, 63] in their communications. Most Level 4 studies involved real or virtual patients as part of the assessment process [117, 120]. Kleinsmith et al. [117] noted that responses to virtual patients tend to be more empathetic than those to simulated patients.

### Enablers and barriers for successful curricula

Table 7 provides a summary of the major enablers and barriers to implementing a successful curriculum.

**Table 7:** Enablers and Barriers to a Successful Curriculum

**Barrier**	**Studies**
Insufficient time for training	[7, 42, 44, 55, 70, 72, 79, 81, 83, 85, 97, 100, 101, 105, 125, 128, 133, 143]
Participant resistance to curriculum	[84, 88, 111, 115, 125, 126, 128, 150, 151]
Inexperience among participants with empathetic communication prior to curriculum	[59, 89, 91, 128, 149, 151]
Stressors outside curriculum affecting participant performance	[5, 41, 97, 148, 151]
Poor role modelling outside of curriculum time	[13, 80, 83, 84, 115, 118, 124, 126]
Lack of resources	[7, 62]
Lack of incentive for participants	[5, 118]
**Enablers**	**Studies**
Adequacy of financial support	[2, 3, 6, 12, 15, 19, 38, 43–45, 50, 54, 56, 59, 61, 64, 70, 71, 75, 77, 78, 80, 84, 96, 100, 106, 113–115, 117, 120, 138, 145]
Timing of curriculum was convenient for participants	[47, 55, 63, 130, 151]
Participants were motivated	[77]
Participant exposure to role models outside of intervention	[16, 58, 62]

## Discussion

### Stage 6 of SEBA: Discussion synthesis of SSR in SEBA

In answering its primary and secondary questions, this SEBA guided review provides a number of key insights.

Our findings suggest that empathy may be described as a “*physician’s recognition and self-regulated cognitive, affective and behavioural* response *to a patient’s, family member’s, caregiver’s and/or a healthcare professional’s distress. This response does not conflate and confuse the patient’s, family member’s, caregiver’s and/or a healthcare professional’s distress with the physician’s own experiences and situation.”* It is also apparent that empathy may be nurtured by building upon the individual’s innate ability to respond to the perceived state of mind, emotion and perspective of the other person. This process of nurturing empathy appears to occur in stages.

Stage 1 involves an introduction to concepts of empathy [4, 12, 115, 119]. These sessions are often in the form of didactic teaching sessions and discussions [7, 90].

Stage 2 acknowledges different learning styles [63] and offers a combination of teaching modalities to provide a holistic approach to nurturing empathy. This includes role play and simulations to practice communication skills [7, 15, 42, 44, 46, 51, 77, 85, 105, 107, 111, 115, 116, 118, 121, 123, 127, 142, 144] in a safe environment to share opinions and observations freely [7, 9, 44, 62, 115, 147].

Stage 3 involves debriefs and personalised, appropriate, specific, timely, actionable and holistic feedback [2, 38, 98, 125]. Reflective exercises [2, 38, 98, 125] and facilitated group discussion are used to explore learning points and experiences [52, 53, 66, 105, 115, 150] and to promote interprofessional education [7].

Stage 4 acknowledges the need to apply interpersonal and empathetic communication skills [44, 118, 142, 144] to elicit a holistic history from the patient [7, 105, 111]. This stage also acknowledges the shortfalls and inaccuracies posed when using simulated and virtual patients [80, 117, 120]. This stage also includes debriefs and feedback [2, 38, 98, 125], reflective exercises [2, 38, 98, 125] and facilitated group discussions [52, 53, 66, 105, 115, 150]. These methods should emphasise on building and bolstering the learner’s confidence and skills when communicating with patients.

Here the notion that external factors – such as practice culture, educational setting, clinical specialities, prevailing sociocultural norms, professional and practical considerations and regnant sociocultural, healthcare and educational systems – also impact empathetic responses suggests that these responses may vary in different circumstances. In addition, evidence that intrinsic motivations are informed by the physician’s demographic, historical, socio-cultural, ideological and contextual circumstances suggests that empathy is also a sociocultural construct demanding a personalised approach when nurturing and assessing empathy in physicians.

Acknowledgment that there are stages to empathy training that are influenced by contextual factors as well as innate considerations underscores the need to develop a ‘spiral curriculum’ [156] where each step repeatedly builds on prior knowledge and skills (horizontal integration) as more complex competencies are introduced and assessed in various settings. This spiralled approach must also be personalised and frequently assessed to inform effective nurturing of empathy. This underlines the need for personalised micro-competencies and general milestones to ensure that physicians are effectively supported in a manner appropriate to their abilities, needs and circumstances as well as the educational goals and training context. Personalised micro-competencies depend on the physician’s training, knowledge, skills, experience, motivations and circumstances, thus underlining the importance of assessing each physician’s individual needs so as to shape training and proffer appropriate support. General milestones relate to common expectations placed upon all physicians at each stage of their training and allows due consideration of the contextual aspects of empathy. The presence of general milestones underlines the need for different stages of the program to be carried out at a period in which there is relevant clinical training (vertical integration). This is so that the process of nurturing empathy occurs at a time where the physician can best appreciate its relevance to their practice and role and thereafter apply their new skills under supervision before doing so independently.

The need for horizontal and vertical integration within the spiral curriculum and the presence of personalised micro-competencies and general milestones underscore the need for efforts to nurture empathy to be integrated into a formal curriculum. The formal curriculum will also facilitate holistic and longitudinal assessments with clear opportunities for targeted and timely intervention and remediation before the physician lags too far behind. In addition, being integrated into the formal medical curricula allows for ‘protected time’ allocated [5, 41, 97, 148, 151] for training both learners and faculty members overseeing and conducting the curricula [7, 42, 44, 55, 70, 72, 79, 81, 83, 85, 97, 100, 101, 105, 125, 128, 133, 143].

However, a general lack of evidence for the efficacy of various assessment tools in the prevailing literature, the different foci of empathy training, and their stage sensitive nature renders the selection of an appropriate assessment tool a challenging task. This is especially so as different tools fundamentally pivot on different facets of empathy’s diverse conceptualisations. For example, the IRI focuses on measures of both cognitive and affective empathy [157] whilst the Empathy Scale focuses only on the cognitive [158] and the Balanced Emotional Empathy Scale only on the affective [159]. As a result, curriculum developers should ideally consider an amalgamation of assessment tools employed along the spiral curriculum, with assessment outcomes ‘stored’ in longitudinal portfolios to ensure transparency and rigorous follow-up across tutors and practice setting. Multi-source assessments of individuals and program outcomes will allow for changes in practices and attitudes [5, 41, 97, 148, 151] and will facilitate the inclusion of holistic assessments from assessors and observers [160] that ought to capture contextual considerations impacting progress and practice.

## Limitations

Whilst SEBA offers an evidence based, comprehensive, reproducible and transparent approach to reviews across a wide range of settings and socio-culturally informed concepts, it is a resource intensive approach as SEBA requires at least three independent teams to perform the Split Approach and Funnelling Process appropriately. Concurrently, whilst the SEBA guided review has provided a number of new insights, reliance upon the expert team may be time consuming as it draws out the various SEBA stages.

In addition, its comprehensive approach does not circumnavigate other limitations such as the exclusion of publications that were not published in or translated into English. This is particularly important given that the concept of empathy is culturally informed. With 77% of the included articles conducted in a Western population, there is a significant risk that the concepts delineated here may not truly reflect how it is conceived in other parts of the world.

Further context specific elucidation of concepts of empathy in physician-patient relationships [1, 2, 4], on patient outcomes [3, 4, 12, 121] and satisfaction [2, 5, 15, 133], physician burnout [10, 11, 68], emotional exhaustion [161], professional satisfaction [6, 7, 12, 60], and clinical competence [4, 8, 9, 110] are required particularly when it appears that contextual considerations surrounding empathy impact behaviour and motivation.

Lastly, large variations in assessment methods were employed across the studies, making it difficult to compare outcome measures of nurturing empathy.

## Conclusion

In answering its primary and secondary research questions, this review advances a more holistic understanding of empathy and proffers a stage-wise framework to guide the design of a formal, multimodal, longitudinal and spiralled program to nurture and assess empathy in medical education.

Whilst it is clear that assessments of empathy need to be improved if empathy is to be effectively nurtured, it is also evident that use of a mix of tools over each stage of the nurturing process requires the employ of portfolios. Portfolios replete with reflective entries and accounts of critical incidents will help assess wider and longitudinal influences upon the learner, including why and how these experiences may have affected their practice and their professional identity. It is evident that pragmatic and efficacious use of portfolios and empathy’s potential links with professional identity formation deserve closer scrutiny in future research endeavours.

## Supplementary Information

**Additional file 1.** PubMed Search Strategy.

**Additional file 2.** Tabulated Summaries.

## Data Availability

All data generated or analysed during this study are included in this published article and its supplementary information files.

## Abbreviations

SEBA: Systematic Evidence Based Approach
NUS: National University of Singapore
YLLSoM: Yong Loo Lin School of Medicine
NCCS: National Cancer Centre Singapore
PCC: Population, Concept and Context
PICOS: Population, Intervention, Comparison, Outcomes, Study Design
JSE: Jefferson Scale of Empathy
IRI: Interpersonal Reactivity Index
QCAE: Questionnaire of Cognitive and Affective Empathy
SEI: Social Empathy Index
SP: Simulated Patients
