# Supplementary material for: A systematic scoping review of approaches to teaching and assessing empathy in medicine
Source: BMC Med Educ. 2021 May 22;21:292. doi: 10.1186/s12909-021-02697-6 (PMC8140468; doi:10.1186/s12909-021-02697-6)
Supplement: Supplementary file 1 — Additional file 1. PubMed Search Strategy. [file 12909_2021_2697_MOESM1_ESM.pdf]

Supplementary File 1. PubMed Search strategy

Database	Search terms
**Pubmed**	("Empathy"[Mesh] OR empath*[TIAB]) AND (“Medicine"[Mesh] OR Medicine[TIAB] OR “medical student”[TIAB] OR “medical students”[TIAB] OR doctor*[TIAB] OR physician*[TIAB])
