# Supplementary material for: A systematic scoping review of approaches to teaching and assessing empathy in medicine
Source: BMC Med Educ. 2021 May 22;21:292. doi: 10.1186/s12909-021-02697-6 (PMC8140468; doi:10.1186/s12909-021-02697-6)
Supplement: Supplementary file 2 — Additional file 2. Tabulated Summaries. [file 12909_2021_2697_MOESM2_ESM.pdf]

Supplementary File 2. Tabulated summaries

Author	Year	Title	Background	Theoretical Approach and Methods	Main empirical findings	Insights drawn
Afghani et al.	2011	Medical students' perspectives on clinical empathy training	Empathy has benefits for patients and physicians alike but a decline in empathy in medical school has been reported in several studies. It is still challenging to include empathy training in formal curriculum.	Medical students participated in a 4-year longitudinal curriculum emphasizing on the patient-doctor relationship, communication skills and empathy. 157 Year 3 and Year 4 students responded to a survey regarding this intervention.	81% felt that their empathy had increased or stayed the same during the training. Students felt that the behavioural component should be emphasised the most during training, followed by moral, cognitive then emotive component. Time pressure and a lack of good role models were reported as the 2most common barriers to nurturing empathy. One-to-one, small groups discussions with preceptors, accompanying patients to clinics, being hospitalized as patients and interpersonal skills workshops were ranked the highest.	Majority of students were satisfied with their training. More attention should be given to help students translate the theoretical understanding of the patient's experience to specific behavioural responses. Topics on specific stressful situations require more attention.
Ahmad et al.	2005	Do informal interviews improve medical student empathy with the elderly?	One survey of medical students demonstrated that only 4% indicated a strong interest in working with older patients. The undergraduate educational goals of many geriatrics societies list appropriate attitudes toward looking after older patients along with knowledge and skills. Inpatient experiences do not	582 students participated in informal interviews of the elderly. This is a qualitative study utilising structured & descriptive analysis of students' essays after completion of interviews.	1) The themes that were targeted in the design of the task, such as expectations regarding aging, independence, and health were well-identified with above-average scores (on a five-point Likert scale). 2) This activity was very acceptable to the students, with many noting that they	Asking students to interview community-dwelling and to report in a structured report format is a very acceptable activity to complement acute, inpatient experiences and allows important themes to be consistently identified by students, which often leads to new insights about older persons.

			necessarily improve attitudes toward older persons.		had gained new insights into older people.	
Ahmadzadeh et al.	2019	Does watching a movie improve empathy? A cluster randomized controlled trial	Because of the beneficial effects of empathy in various outcomes of physician-patient encounter, many studies have attempted to improve empathy in health professionals and students. This is especially important because many studies have reported that empathy score of medical students decreases with increasing years of education.<br>Watching films or movies has been successfully used both as a method to improve empathy and a method combined with another educational method to improve sustainability of the increased empathy.	42 medical students participated in a 3h communication skills training, 23 medical students participated in a 2h screening of the movie 'The Doctor', 22 medical students participated in a 2h screening of the movie 'The Doctor' followed by a 3h communication skills training the next day, with 46 medical students participating in a control group.<br>This is a pretest-posttest quantitative study with a control group utilising the Jefferson Scale of Empathy, Student Version.	All the active intervention groups showed an increase in JSE score, but group D (control group) did not show a significant change. The increase was more prominent in groups B and C. However, at 1-month follow up, JSE score decrease was observed in all three active intervention groups and was more pronounced in group B (film only).	All the three interventions (communication skills workshop, watching the movie, and workshop plus watching the movie) have an immediate positive effect on empathy scores of medical students compared to control group. However, watching the movie seemed to increase the immediate effect and participating in the workshop tended to decrease the decline of the score during the follow up and so appeared to improve the sustainability of the effect of the intervention.
Airagnes et al.	2014	Appropriate training based on Balint groups can improve the empathic abilities of medical students: A preliminary study	Physician's empathic abilities are essential to build a strong doctor–patient relationship, which is known to improve treatment adherence and clinical outcomes. A decline in empathy throughout medical school has been observed mainly at the end of first and third year.<br>Balint groups are specifically designed to help	60 fourth-year medical students participated in the Balint group, with 34 participating in the study. 379 other student participated in other activities, with 129 participating in the study as a control group.<br>This is a pretest-posttest quantitative study with a control group utilising the Interpersonal Reactivity	Students in the Balint group exhibited an enhanced Empathic-approach, and demonstrate a better ability to take into account this affective dimension of the doctor–patient relationship when they were faced with a clinical situation including relationship difficulties, like the case of patients with borderline personality traits.	The authors' findings support a more optimistic view on the issue of empathy in medical curriculum and encourage assessing training initiatives designed at helping young medical students to take into account the emotional component of doctor–patient relationship, despite their still lacking clinical experience.

			health-professionals and medical students in developing their empathy skills in order to reduce interpersonal difficulties.	Index (IRI) and an ad hoc 8-item questionnaire aimed to assess their reactions in response to two written case-reports		
Alexander et al.	2015	Teaching an experiential mind-body method to medical students to increase interpersonal skills: a pilot study	The medical literature suggests that physician empathy is based primarily on a combination of reflective listening and mindfulness, and medical student empathy has been shown to decrease over time.	6 students were randomly selected out of 50 to take part in an inner relationship focusing intervention elective focusing on empathic listening. Intervention and control group completed a pre and post course survey measuring their perceived self-awareness and comfort with clinical interactions.	Only the difference between pre and post intervention score of the "comfort with relating recurring non-organic symptom might be related to an issue in that patient's life" component reached statistical significance. Overall comments about the programme were positive.	The inner relationship focusing simple intervention that has a potential benefit in medical education and may help with reducing burnout among students as suggested in other studies. This novel intervention may serve as preliminary data for further investigations in the future.
Batt-Rawden et al.	2013	Teaching empathy to medical students: an updated, systematic review	Empathy is beneficial to patients. However, there has been debate on whether there is a decline of empathy over the years among students and professionals and disagreement on the validity of self-report measures as accurate. This systematic review aims to update and systematically expand the most recently published literature on empathy-enhancing education interventions for undergraduate medical education.	A search was done from Jan 1 2004 to March 19 2012. The concepts of undergraduate medical and empathy was defined using a combination of controlled vocabulary terms and keywords.	Empathy interventions can successfully nurture empathy in undergraduate medicine and are well received by participants.<br>The empathy decline was reported in many of the studies included.<br>Specific characteristics (e.g. higher baseline scores, Asian ethnicity, specialty choice) were associated with greater improvements, suggesting that certain interventions should target certain groups.	More work can still be done to translate the theoretical empathic skills developed through interventions into behaviour, hence second or third person assessments can be considered.<br>It remains to be understood on whether the respective domains of empathy influenced by the teaching methods are valued by assessors and patients. The clinical impact of such interventions should be investigated further.<br>More rigorous, large and properly controlled longitudinal studies are still lacking and necessary.

Bayne	2011	Training Medical Students in Empathic Communication	The quality of the doctor-patient relationship has an impact on both the physician and the patient. Studies have shown a decline in empathy throughout training, yet studies have shown that empathy can be taught. Previous studies have shown that group process, facilitated by a mental health professional, can improve the empathic ability of medical students and doctors.	22 third year medical students from a medical school in southeast Virginia took part. The students participated in training involving didactic lectures on empathy and communication skills. Role plays and group discussions were also used to facilitate learning. SP interviews before and after the intervention was used to evaluate the student's empathy levels with the CARE measure.	1) Participant scores on the CARE measure increased across groups after the training<br>2) Students reported the training helped them improve their communication skills<br>3) Students reported on some of the stressors for empathetic behaviour that they face.<br>4) Students reported a need to protect themselves from the "inner world" of the patients	The implemented training was useful in improving student empathy communication skills as measured by patients. Group discussion, roleplays, and didactic content are all effective at teaching various aspects of empathy for students.
Bays et al.	2014	Interprofessional communication skills training for serious illness: evaluation of a small-group, simulated patient intervention	Good patient-clinician communication has been shown to promote better patient outcomes. However, despite its importance, communication skills training have not be shown to cause behavioural change. Trainees report poor communication and infrequent role-modelling. This report aims to investigate the effect of an experiential communication skills training on internal medicine trainees' and nurse practitioner students' ability to communicate bad news and express empathy.	477 participants took part in an interprofessional communication skills training workshop (Codetalk) involving facilitated didactic sessions, roleplay and reflections.<br>This is a quantitative pretest-posttest study. Participants completed an SP interview during the first and last workshop sessions. Coders rated the participants using SPIKES (a six-step protocol for communicating bad news) and NURSE (on verbal empathic expression). There was	1) Trainees had statistically significant improvements in 4 of the 6 SPIKES components.<br>2) Trainees had statistically significant improvements in 4 of the 5 NURSE components: naming, respecting, supporting and exploring.	Small-group communication skills training can promote measurable changes in communicating bad news and expressing empathy.<br>The lack of association between improvement and participant characteristics suggest that this intervention can be effective for all disciplines and levels of experience.<br>The development of the intervention on one site and successful implementation on another suggests that it can be exported successfully.

				complete data from only 128 residents and 17 nurses.		
Bentley et al.	2018	Relational Mindfulness for Psychiatry Residents: a Pilot Course in Empathy Development and Burnout Prevention	Empathy builds on self-monitoring and self-care, but this is often overlooked in medical training. Overidentifying with the emotions of others might also stunt empathy development. Mindfulness training could teach mental habits important for empathic attunement to others and increase healthcare providers' capacity for empathy by reducing stress and burnout.	7 PGY1s participated in a eight 1.5-h mindfulness and empathy training (4 sessions each). Outcomes were measured with the Helpful Responses Questionnaire (HRQ), MBI-HSS and a Learning Experiences Questionnaire (LEQ) developed by the authors.	Quantitative:<br>There was significant positive change in HRQ and a downward trend on the MBI-HSS.<br>Qualitative:<br>- increased awareness of their cognitive and/or emotional experiences<br>- better self-care and patient care<br>- expect more empathy in future clinical work<br>- paperwork, pre-set approach to patients, high patient volumes and burnout were barriers to integrating the skills into practice	This course was effective (though limited) in improving empathy.
Bergstresser	2017	Empathy in medical students: Exploring the impact of a longitudinal integrated clerkship model	Much work has been done to investigate the nature of empathy and this has led to the development of many tools to measure empathy. Empathy is an essential component of patient care with benefits for both the patient and physician, and many studies have attempted to increase empathy among medical students and doctors. The traditional longitudinal clerkship model of medical education has been adopted around the world, yet few	This study looks at archival data from a medical school containing JSPE and PPOS scores of 186 medical students collected at each year of their clinical clerkships.	1) While the baseline empathy of the students in this study were the same as in other studies, at the end of 3rd year, the empathy levels were significantly higher than other studies.<br>2) No associations between empathy and specialty choice were found in this study.<br>3) Empathy levels increased from 2nd to 3rd year then decreased in 4th year.	The longitudinal clerkship model might have mitigated the erosion of empathy among medical students. Those who valued patient-centredness also tended to have higher empathy, thus suggesting that the clerkship model can improve patient-centredness and lead to less decrease in empathy.

			studies investigate the impact it has on student empathy.			
Blanco et al.	2013	Efficiency is not enough; you have to prove that you care: role modelling of compassionate care in an innovative resident-as-teacher initiative.	Current literature highlights the need to promote patient-centredness among the medical community. However, there is a paucity of effort aimed at training medical educators on teaching humanistic care.	41 residents took part in a 9-month training programme aimed at teaching compassionate care through role modelling. A mixed methods programme evaluation design was developed to assess outcomes of the training. This involved Pre/post intervention SP interviews, JSPE, self-assessment of relationship-centred skills, journal reflections, presentations, and programme evaluation.	1) Self-score of relationship-centred skills as caregivers increased significantly post-intervention.<br>2) JSPE and SP evaluation scores also increased but these were not statistically significant.<br>3) Resident journal entries provided some barriers to relationship-centred care.<br>4) Overall impression of the programme was positive.	Support and validation of the effort made to provide compassionate care is important and can help to combat the erosion of empathy.
Bombeke et al.	2011	Medical students trained in communication skills show a decline in patient-centred attitudes: an observational study comparing two cohorts during clinical clerkships	Patient-centredness and effective communication skills have been identified as an essential component for better patient care. While interventions in both have been attempted, few look at the long-term retention of the skill learnt.	A comparative cohort study was conducted involving 2 cohorts of medical students, one with 55 students and the other with 45. One cohort received a 5-year curriculum with communication skills while the other received a traditional curriculum without communication skills training. Both cohorts were assessed at the start and end of their respective clinical clerkships using the	1) Patient-centredness and empathy remained stable in the control group but decreased slightly in the intervention group<br>2) Attitudes towards communication skills learning remained stable in the control group but decreased in the intervention group<br>3)	1) The ideal environment for communication created in communication skills training created a big gap in between the training ground and the clinical setting. After experiencing the clinical setting students were more averse to what trainers were teaching them.<br>2) Current measures to assess outcomes of training are not nuanced enough to quantify the changes in communication skills among medical students.

				Doctor Patient Scale, Social context scale, Leeds Attitude to Concordance Scale, JSPE, and Communication Skills Attitude Scale.		
Bond et al.	2013	Embodied health: the effects of a mind-body course for medical students	An effective career in medicine requires technical expertise and competency coupled with high levels of empathy, personal well-being, and connectedness to others. In addition, research indicates patients' perception of physician empathy correlates with levels of satisfaction and compliance with medical recommendations. Ironically, medical students report that the demands of medical education such as a heavy workload, abundant stress, and a sense of competition tend to increase burnout and decrease the ability to connect with patients. For example, overall empathy levels drop as burnout and suicidal ideation temporally rises among students. However, research on yoga and mindfulness meditation suggests these practices may improve psychological wellbeing, such as by	27 first-year and second-year medical students participated in a 16.5h mind-body course (yoga, mindfulness and an educational component) This is a pretest-posttest mixed methods study utilising the Jefferson Scale of Physician Empathy, the Cohen's Perceived Stress Scale, the Self-Regulation Questionnaire, the Self-Compassion Scale, and an essay reflecting on any insights or how the elective has influenced them.	1) Common themes found in students' end-of-course essays included increased mindfulness and its positive effect on self-regulation; a feeling of community in an otherwise competitive environment with an increase in empathy for others; positive effects of increased mindfulness as reflected by increased self-regulation; a decrease in stress level; and increased competency and interest in suggesting mind body practices that could benefit patients<br>2) Intervention had a statistically significant positive effect on students' self-regulation and self-compassion.<br>3) There was also a favourable change in empathy and perceived stress, but these changes did not reach statistical significance.	A semester-long mind body course for medical students was beneficial for students' self-regulation and self-compassion, while empathy and perceived stress also experienced favourable changes (but did not reach statistical significance).

			decreasing perceived and physiological measures of stress			
Bonvicini et al.	2009	Impact of communication training on physician expression of empathy in patient encounters	Despite evidence which demonstrate the importance of physician empathy to patients, studies reported that it is often lacking in medical encounters. However, there is a dearth of evidence on how an educational intervention for physicians can improve their response to empathic opportunities in patient interactions, while many of the studies on physician communication behaviours only used single evaluation measures. This study evaluates the effects of an educational intervention on physician-expressed empathy using audiotaped physician-patient interactions from a large randomized control trial.	Out of 160 doctors, 80 had undergone an three 6h communication workshops over 11-months. This is a pretest-posttest quantitative study. The audiotapes were taken at baseline and post-intervention.1800 audiotaped interactions between all 160 doctors and their patients were coded with the Global Rating Scale. 232 randomly sampled audiotapes were then recoded using the Empathy Communication Coding System (ECCS).	1) There was a significant increase in global empathy scores among the intervention group, while there was no change in the control group. 2) There was a significant increase in physician empathy response scores (ECCS) while there was no significant change in the control group.	Third-party measures may not fully account for non-verbal communication in empathic expression. Nonetheless, this intervention supports that repeated sessions with follow-up reinforcements are important to sustain learned skills and reinforce behavioural change. Multi-modal learning strategies are important to effectively change physician behaviour.
Bunn & Terpstra	2009	Cultivating empathy for the mentally ill using simulated auditory hallucinations	Empathy for patients is a difficult attribute to learn and plays a major role in the doctor-patient relationship. The ability to appreciate patients' emotions and express this emotional awareness improves clinical outcomes, professional satisfaction, and patient adherence to medical	100 medical students participated in an auditory hallucination simulation with 50 other medical students acting as a control group. This is a pretest-posttest quantitative study with a control group utilising the Jefferson Scale of	After intervention, students' JSPE scores had a statistically significant increase, whereas students in the control group revealed no significant difference in their empathy scores.	Students who listened to the simulated auditory hallucinations had an increase in their overall empathy score. Schools may consider utilising specific interventions such as a simulated auditory hallucination to help students increase their empathy for the mentally ill.

			recommendations, and is believed to significantly improve patient satisfaction. Although the benefits of demonstrating empathy for the patient have been well documented, it is troubling to note that empathy declines during medical school and residency.	Physician Empathy, Student Version		
Cai et al.	2019	Can I Get a Suggestion? Medical Improv as a Tool for Empathy Training in Obstetrics and Gynaecology Residents	Empathy is a key aspect of the patient-physician relationship. Physician empathy has been associated with higher levels of patient satisfaction, adherence to medical recommendations, and improved clinical outcomes. However, as medical professionals progress through their training, empathy tends to decrease. Methods have been developed over the last few years to combat this, including incorporating the humanities into medical education.	22 OBGYN residents participated in a 1h improv workshop.<br>This is a pretest-posttest quantitative study utilising the Jefferson Scale of Physician Empathy	There was a significant difference in the mean JSPE score immediately post-intervention compared to pre-intervention, but this difference was not statistically significant at the 1-month, 3-month, or 6-month follow-up	OB-GYN residents in this study had a small increase in empathy immediately after a medical improv workshop, although its effect diminished over time.
Canales et al.	2019	Humanistic medicine in anaesthesiology: development and assessment of a curriculum in humanism for postgraduate anaesthesiology trainees	The need for a formal humanism curriculum in medicine becomes more critical considering the increased use of technology which might dilute interactions between patients and their healthcare providers. The authors developed a humanism	10 first-year anaesthesiology residents participated in a formal humanism curriculum involving interactive workshops, simulation sessions, formal feedback and patient immersion experience.<br>Patients rated the	1) Residents were rated to be more empathetic and professional and overall satisfaction has improved. Patients reported to have lower anxiety and a lower pain score after training.<br>2) Residents empathy ratings reported by the JSE-HP increased.	This study supports that a formal humanism curriculum can improve empathy, professionalism and patient-reported outcomes. The authors argue that the 'hidden curriculum' in medicine should be given more focus and cultivated with good role modelling. Teaching

			curriculum as part of formal postgraduate anaesthesiology education.	participants using the JSPPPE, perceived resident professionalism questionnaire, patient satisfaction questionnaire, VAS-A and PNRS pre-test and post-test. Participants were also administered the JSE-HP pre-test and post-test. Outcomes were evaluated using the Kirkpatrick model of training and assessment.		humanistic skill can have a major impact on patient-centred care and improving the personalisation in medicine for physicians.
Cataldo et al.	2005	Association between Balint training and physician empathy and work satisfaction	Balint group discussions are now offered throughout the world and are part of the curriculum in nearly half of all family medicine residency programs in the United States. In today's rapidly changing world of medicine and health care systems, physicians require skills to prevent burnout and improve job satisfaction while preserving a professionally rewarding doctor-patient relationship. Although existing data is encouraging, there remains a need to document the benefits and outcomes of Balint groups in family medicine residency programs.	113 family physicians participated in a weekly 1h Balint group session for 2 years, with 69 other family physicians acting as the control group. This is a quantitative study with a control group utilising the Jefferson Scale of Physician Empathy, and physician Work Satisfaction Survey.	1) There was no statistically significant difference in JSPE scores between Balint attendees and Balint nonattenders.<br>2) There was no statistically significant difference in Work Satisfaction Survey scores between Balint attendees and Balint nonattenders.<br>3) A significant difference was found between the two groups in their desire to choose the same medical specialty again if graduating from medical school today, with more Balint attendees stating that they would choose family medicine as a career specialty as compared to Balint nonattenders.	This study did not find an association between Balint training and physician empathy, overall work satisfaction, or financial compensation. However, the study did demonstrate that graduate physicians who chose to continue participation in Balint training were more likely to choose family medicine as a career specialty again.

Chen et al.	2018	Teaching Empathy: the Implementation of a Video Game into a Psychiatry Clerkship Curriculum	Empathy is essential to the patient-physician relationship and is beneficial to patients and physicians. However, empathy typically decreases in medical training. Video games have been shown to be an effective teaching tool. This study looks at the possible effectiveness of interactive video games as a tool to teach empathy to third-year medical students.	84 Year 3 medical students played the video game "That Dragon, Cancer". This is a pretest-posttest quantitative study using the first 5 questions of the JSPE to assess students' empathy. They also gave feedback on the intervention.	There was a statistically significant improvement in the first five questions of the JSPE. Majority of students found the video game more effective in learning empathy than didactic methods, and that they preferred video games.	Video games can be an effective tool to teach empathy. Students also enjoyed it and found it more effective than didactic methods despite some complains of the game mechanisms and gameplay. Females and males can be equally receptive to video games as a learning tool, probably due to its educational intent. Video games and the virtual setting have potential in medical education.
Chinai et al.	2016	The ABCs of empathy	Empathy has been shown to be declining amongst healthcare providers despite its importance and burnout affects empathy.	This conference abstract describes an intervention to increase empathy and decrease burnout. The ABCs of empathy focuses on mindfulness, patient-centred communication and reflection. 10 intervention group residents rated their satisfaction level of the curriculum on a 10-point Likert scale.	All the components of the curriculum were rated 7.1 to 8.8 on average. 9/10 residents recommend it to other residents.	This curriculum can be easily incorporated into residency conference didactics.
Chunharas et al.	2013	Medical students themselves as surrogate patients increased satisfaction, confidence, and performance in practicing injection skill	Injection skill is one of procedural skills usually taught to medical students. Lack of experience and confidence in their ability creates nervousness, anxiety, and leads to poor performance in giving an injection to patient.	116 fifth year medical students were split into a control and intervention group where there were taught injection skills using mannequins. The intervention group was then allowed to voluntarily experience the	Before giving injections to children, 80.7% of students in the control group and 59.4% of those in the intervention group had high empathy for children. After the injection, this number rose to 84.4% in the intervention group but	Being a surrogate patient allows students to develop better professional attitudes to patients by expressing more empathy.

				injection. Both groups were then allowed to give injections to children as part of their clinical paediatric posting.	decreased to 74.4% in the control group.	
Cinar et al.	2012	Communication skills training for emergency medicine residents	Emergency departments (EDs) have unique dynamics about doctor–patient communication. Emergency physicians typically must obtain information from overstressed patients who they meet for the first time, make fast decisions regarding diagnosis and treatment, give information to patients and relatives, and communicate with the consulting doctors and other medical staff. They must also manage stressful situations that are routine in EDs. They are also expected to establish an appropriate line of communication with special groups of patients, for example, patients with altered consciousness and those who have attempted to commit suicide or are psychotic. Therefore, effective communication is a critical skill for successful management in EDs.	20 emergency medicine residents participated in a 9h communication skills training.<br>This is a pretest-posttest quantitative study utilising the Empathy Quotient, the Communication Skills Inventory, and a patient satisfaction survey.	1) Mean total communication skills score of residents increased after the training.<br>2) Mean empathy score rose but this was not statistically significant.<br>3) Patient satisfaction scores also rose after the training.	Participation in a communication skills training program was associated with improved communication skills of emergency medicine residents, and increased patient satisfaction and decreased complaints.
D'Souza et al.	2019	Effect of a Single-Session Communication	An empathetic physician, proficient at perceiving non-verbal cues, may be better at	43 medical students participated in a 2h communication skills	1) The JSE score significantly improved immediately after the	The empathy scores of the second-year medical students improved significantly after a

		Skills Training on Empathy in Medical Students	discerning patient's concerns and putting them at ease, which aids exploration in a clinical setting. This facilitates a deeper understanding of the patient's experience, enhances accuracy of diagnosis, and enables effective treatment. These physicians, perceived to be more caring, allay patients' anxiety and promote a feeling of empowerment, thus, improving patient satisfaction, adherence to treatment and clinical outcome. Being empathic also carries certain benefits for the physician such as a sense of wellbeing, lower burnout, and better job satisfaction. Several studies have shown a decline in empathy as medical students progress from the first year towards the final year of medical education. The decline in empathy has been ascribed to several factors, some which include the expectation to learn large volumes of information in limited time frames, lack of sleep, emotional fatigue and burnout, as a method of coping, lack of role models, and the belief that emotions	training, with another 38 students participating in a control group.<br>This is a pretest-posttest quantitative study with a control group utilising the Jefferson Scale of Empathy-Student version	intervention but dropped at follow-up, 3 weeks after the intervention, though this drop was not statistically significant, compared to the baseline.<br>2) At the follow-up assessment, the mean JSE score of the intervention group was higher than the control group though this difference between the groups was not significant.	single-session training on empathetic communication skills. However, the effect was not sustained over a period of time.

			interfere with clinical decision making Whether empathy can be taught has been much debated.			
DasGupta et al.	2004	Personal illness narratives: using reflective writing to teach empathy	Narrative medicine, particularly reflective writing has been shown to improve empathy	16 Year 2 students participated in a reflective writing exercise on a personal illness during a humanities seminar. 11 students completed an evaluation form with 7 questions. The responses were analysed using content analysis and thematic analysis	It was well perceived by participants and they highly recommend it.<br>Evaluation revealed that they had an increased sense of regard and empathy for their patients.	Sharing of emotional and physical vulnerability is rare in medical school but has received positive reactions. This intervention helped them to articulate and examine their own feelings. Personal illness narrative writing helped participants to witness, interpret and translate their own and other's experiences to integrate similarities between their own personal experiences and those of a patient and understand themselves and others better.
Deen et al.	2010	Points of contact: using first-person narratives to help foster empathy in psychiatric residents	Empathizing with patients is key to becoming a good clinician. Various approaches have been attempted to foster empathy. Writing narratives can be powerful and effective in fostering empathy.	One PGY1 resident wrote narrative pieces of 3 patients of her own choice in a community mental health clinic. To help the resident learn about her feelings about the patient, the supervisor and resident examined the style of writing, choice of words, and story line.	The resident felt that discovering "points of contact" (i.e. Clues about the patient which helped the resident to empathize with the patient's subjective experience) helped her to connect to her patients and promote empathy.<br>The process improved her interviewing skills. Feeling the patient's feelings helped her to feel more empathy toward the patient.<br>The supervisor also noticed increased empathy towards the patient	Writing imaginative narratives help one resident to connect to emotionally distant patients by imagining their points of view.<br>Narratives promotes self-reflection and countertransference and provide time to reflect about their patients.

Delacruz et al.	2017	Take the HEAT: A pilot study on improving communication with angry families	Effective communication skills are important for producing better patient outcomes, and studies have shown that these skills can be nurtured. However, there is a lack of training in most residency programmes.	33 paediatrics residents took part in the intervention involving a e-learning module that focuses on communication skills. Assessment of the participants was done using SP interviews before and 1-6 weeks after the intervention was completed. The assessment rubrics was based on the novel HEAT methods.	Overall performance significantly improved after the intervention (27 out of 33 demonstrated an overall improvement.)	There are lapses in the current training curriculum in providing doctors with the tools to communicate with angry patients. Relying on the innate communication skills of doctors is insufficient, therefore more focus should be placed on developing specific areas of communication for doctors.
Deloney & Graham	2003	Wit: using drama to teach first-year medical students about empathy and compassion	Empathy is an essential component of patient care with benefits for both the patient and physician. Studies have shown a decline in empathy throughout training, yet medical training largely ignores this. Recent studies have shown increasing interest in using virtual patients to improve empathy.	Medical students watched the play Wit and took part in discussion after it. Within 1 week of the performance, students were asked to reflect on their reactions to the play in an email dialogue with their faculty.	Most students enjoyed the play and preferred it over a didactic lecture or reading journal articles. Students realised the potential for their medical education to suppress their empathy and compassion. Students' self-rating of their ability to address a patient's physical pain was found to be higher than their ability to break bad news, discuss treatment plans, control symptoms, and deal with a patient's emotions.	Experiential learning can increase student interest and help them connect abstract ideas with concrete knowledge. Drama can provide the opportunity for self-reflection in a safe environment and can be an effective tool for attitude change. Post-play discussions provided an avenue for sharing emotions and helped to build a community of support among the students.
DelPrete et al.	2016	Medical Students' Attitudes Toward Non-Adherent Patients Before and After a Simulated Patient-Role Activity and Small-	Empathy is just as important as clinical expertise for effective patient care. Studies have observed a sharp decline in empathy from year 1 to four in medical school. Increased	118 Year 1 students participated in the simulated patient-role activity on Type 2 DM. 62 students completed the pre-test and post-test using the JSPE-S. Their	There was no significant increase between pretest and post-test scores.<br>There was no statistically significant difference between the M1posttest score and the M4 class.	The lack of effectiveness of the intervention in the short term suggests that empathy takes months or years to develop and solidify. The JSPE has its limitations in measuring cognitive and

		Group Discussion: Revisited	empathy towards a particular patient group might translate to that towards all non-adherent patients. An unpublished study showed that simulation of Type 2 DM was the most significant in increasing empathy levels, when compared to that of HTN or MDR-TB	results were compared to that of 16 Year 4 students.		emotional empathy independently and empathy is a difficult construct to measure.
Dereboy et al.	2005	Teaching empathy in medical education	Communication skills are important to clinical outcomes and are gradually becoming an important part of medical education in UK and USA.	98 Year 1 medical students participated in a communication training. This is a pretest-postttest quantitative study using the Empathic Tendency Scale (ETS) and Instrument for Attitudes Towards Communication in Medicine (IATCM).	There was no statistically significant change. There was a negative change in the empathy high group and a positive change in the empathy low group, though not statistically significant. There were significant differences before and after training between the negative-positive attitude and high-low empathy groups. More students who had a positive attitude towards communication had higher empathy than those who had a negative attitude.	Appropriate educational methods might help to improve empathic tendency, particularly in low empathy students where it is more important.
Dhaliwal et al.	2018	Reflective student narratives: honing professionalism and empathy	Effective communication and empathy are essential in the doctor patient relationship. While reflective writing has been shown to be useful in nurturing both in western	Medical students took part in a reflective writing workshop. The content of their narratives was analysed based on the ABCDE paradigm.	Students reflected about both positive and negative experiences they had with doctors during their training. Most students enjoyed the reflective writing practice and agreed that it helped them develop patient-	Narrative writing is a feasible and acceptable intervention that promotes reflection and helps to develop students' affective competencies, leading to increased patient-centredness and empathy

			literature, this method is rarely adopted in India.		centred care while teaching them about ethics. Students agreed that narrative writing were useful for teaching core competencies outside of the usual lessons they received. Students reported that they have better insight to communication with patients and understand the role of a doctor better, causing them to feel greater empathy.	
Ditton-Phare et al.	2016	Pilot Evaluation of a Communication Skills Training Program for Psychiatry Residents Using Standardized Patient Assessment	Effective communication skills are important for producing better patient outcomes, and studies have shown that these skills can be nurtured. While interventions to do so have been attempted in the past, few focus on improving communication between healthcare providers and patients with severe psychiatric disorders, despite reports of these patients being dissatisfied with the care they receive.	30 psychiatry residents took part in the ComPsych programme which uses videos, role-plays, and group discussions to improve communication skills. Self-reported confidence in communication and SP assessments were done pre and post training. Participants who did not take part in the training but took the assessments were used as the quasi-control group.	1) Agenda setting was increased, questioning skills decreased<br>2) Empathetic communication skills increased post training<br>3) The control group showed no significant changes in the SP assessment.	In general, the results of this study validate what has been shown in previous studies. However, no gender associations were found unlike in previous studies. While long term effects of the intervention were not studied, a key strength of this intervention is that participants are able to practice what they learned immediately when they return to work and can use in all their patient interactions, and the authors predict that this will improve long term retention of the skills learnt. Therefore, despite the limitations of the study, it serves as a foundation for which future work can be done to improve communication skills.

Dow et al.	2007	Using theatre to teach clinical empathy: a pilot study	Empathy is important for physicians, yet it is difficult into traditional medical education, leading to a paucity of evidence in this field.	20 residents (6 control, 14 intervention) took part in four 90min classroom and workshop sessions focusing on empathy and communication skills. Participants were evaluated at least once before the intervention and at least once in the 4 months after the intervention. Evaluation consisted of a observed primary care clinic visit between the participant and a patient and they were scored using a modified scoring tool normally used by the Theatre department.	The intervention group showed significant improvement across all sub scores. The intervention group still performed better than the control group even after controlling for pre-intervention scores.	A collaborative approach between faculty in Medicine and Theatre can be effective in teaching empathy skills.
du Vaure et al.	2017	Promoting empathy among medical students: A two-site randomized controlled study	Empathy has been shown to be beneficial for patient care but has been documented to decline during medical training. Interventions to improve empathy have mostly targeted communication skills and evaluated by patients. However, none have used a randomized controlled design.	Out of 352 Year 4 students, 176 students participated in 7 weekly 1.5-hour Balint group sessions.<br>Data from 155 students from the intervention group and 144 from the control group was analysed. They were assessed with the CARE scale by SPs during OSCEs and a prettest-posttest JSPE-MS.	1) Mean JSPE scores increased in the intervention group but decreased in the control group.<br>2) There was no significant difference in mean CARE scores between the intervention and control group.<br>3) Men in the intervention group had higher CARE scores than men in the control group, but this was not the case in females.	This intervention might be more efficient for self-reported empathy than externally assessed empathy, both of which are important to the doctor-patient relationship.
Duke et al.	2015	Preserving third year medical students' empathy and enhancing self-	The loss of empathy in medical students is well documented. Small group discussions have been	31 small groups of medical students were added to faculty-facilitated small group	1) GRAS scores increased overall but JSE scores were unchanged.<br>2) Students reported that the	The results suggest that the use of this online platform is well received and can help to preserve empathy levels and

		reflection using small group "virtual hangout" technology	shown to be effective at preventing this.	discussions using Google Hangouts to allow for discussions and self-reflection. Students completed the JSPE and GRAS before and after the course. Anonymous feedback was also collected at the end of the course.	peer group was a valuable experience because of the peer support, opportunity to share challenges in medical training, and the platform for self-reflection that the intervention provided.	provides a platform for students to share their experiences and troubles with like-minded peers.
Esfahani et al.	2014	A pilot randomized controlled trial on the effectiveness of inclusion of a distant learning component into empathy training	Empathy is an essential component of patient care with benefits for both the patient and physician. Studies have shown a decline in empathy throughout training, yet studies have shown that empathy can be taught. Communication skills training have been shown to be effective in improving empathy.	14 first year residents of psychiatry were randomised into 2 groups of 7. One group took part in a 2-day communications skills workshop consisting of lectures and roleplays. The 2nd group watched a video of the workshop and attended the 2nd session of the workshop. An OSCE was used to assess pre-training empathy and 3 months after the training. JSPPPE was used for post intervention.	14 first year residents of psychiatry were randomised into 2 groups of 7. One group took part in a 2-day communications skills workshop consisting of lectures and roleplays. The 2nd group watched a video of the workshop and attended the 2nd day of the workshop. An OSCE was used to assess pre-training empathy and 3 months after the training. JSPPPE was used for post intervention.	1) The 2 day communication skills workshop was effective at improving empathy<br>2) Replacing one of the days of face-to-face workshop with a video recording was not effective at improving empathy<br>3) While face-to-face teachings are effective, further work needs to be done to find alternatives to it.
Farnill et al.	1997	Videotaped interviewing of non-English speakers: training for medical students with volunteer clients	Language barriers can interfere with the provision of care for patients.	60 second year medical students interviewed non-English-speaking background volunteers for 16 one-hour sessions over 7 months, after which feedback and discussion was done. Volunteers could provide anonymous feedback.	1) Volunteers mainly reported a positive experience<br>2) Students reported themselves as more competent in communicating with people from non-English-speaking backgrounds (eg. avoiding jargon and idioms etc.)	The intervention provides medical students with exposure to people of different ethnic backgrounds and can help to increase confidence in interviewing patients from non-English-speaking backgrounds

				Students rated their own competencies at the beginning and end of the course. A (blinded) psychologist rated the interviews to provide an objective assessment of any improvement in interview skills. JSE was used before and after the training.	3) Observer rating of the interviews improved for 39 students, with 2 showing no change and 18 showing worse performance	
Fernández-Olano et al.	2008	Impact of clinical interview training on the empathy level of medical students and medical residents	Empathy in a warm relationship is not hard but becomes more difficult when we feel little warmth or perhaps even aversion toward the patient. In such cases, the student may even wonder if it is mere play-acting, but play-acting is not equivalent to hypocrisy. Rather, we are setting the stage for a habit of solidarity reinforced through repetition. Empathy is a skill that can be acquired by practise and should become a habit. Physician empathy continues to be an unexplored area in medical education. Social change has led to new needs that require changes to develop an 'effective' professional with appropriate skills, including empathetic capacity, a trait considered important in	46 residents and 82 medical students participated in a 25h communication skills workshop, with 20 residents and 55 medical students participating in a control group.<br>This is a pretest-posttest quantitative study utilising the Jefferson Scale of Physician Empathy.	1) After the intervention, there was a statistically significant increase in JSPE score among workshop participants, a difference not observed in the controls who had slightly higher, but nonsignificant, second JSPE scores<br>2) Students were more likely to have improved scores than residents	A communication skills workshop yields a slight improvement of crucial practice importance in subjects' empathy.

			physicians. Hence, encouraging the physician-patient relationship has become important in medical training and led to the definition of new guidelines for theoretical and practical training. There are no conclusive data, however, on the ability of these practises to improve a subject's empathy level, often considered an inherent personal trait resistant to change.			
Fine & Therrien	1977	Empathy in the doctor-patient relationship: skill training for medical students	It has been generally acknowledged in medicine that emotional and psychological functioning greatly influence health, either hindering or hastening the physical recovery process. Yet this affective dimension has been relatively neglected in the teaching of the medical interview with the result that emotionally sensitive information is often lost. Increasingly, there is interest in training the physician to be responsive to the patient's concerns in addition to obtaining factual information, therefore, empathy as a basic interpersonal relationship skill is seen as being	20 medical students participated in a 12h basic interviewing technique class, with 23 other students participating in the control group.<br>This is a pretest-posttest quantitative study with a control group utilising the Truax Accurate Empathy Scale.	Pre-test scores indicate that both the experimental group and the control group were below level two on the empathy scale, but on the post-test measures, the experimental group averaged above level five, while the control group was still functioning below level two.	An examination of the results indicated clearly that those students involved in a systematically planned interpersonal skill training program were significantly more able to respond emphatically to patients than students in a control group exposed only to the regular curriculum.

			appropriately taught along with other medical interview skills.			
Flint et al.	2017	Discussing Serious News	Effective communication skills are important for producing better patient outcomes, and is especially important in palliative care. Studies have shown that these skills can be nurtured, with experiential learning being one of the more effective modalities.	Paediatric residents took part in a communication skills workshop aimed at providing a framework for addressing difficult conversations with opportunities to practice the new skills. A total of 6 workshops were held and each resident completed a survey at the end of the workshop to rate their communication skills.	1) Majority of participants wanted more training after the workshop and were happy with the way the workshops were run<br>2) Residents reported greater confidence in discussing bad news after the workshop	While residents reported receiving similar training in communication skills while in medical school, a lack of continued training and opportunities for practice led to low confidence in delivering bad news. Using bereaved parents as volunteer patients adds to the realism of role play and leads to a better learning experience.
Foster et al.	2016	Using Virtual Patients to Teach Empathy: A Randomized Controlled Study to Enhance Medical Students' Empathic Communication	Empathy is an essential component of patient care with benefits for both the patient and physician. Studies have shown a decline in empathy throughout training, yet medical training largely ignores this. Recent studies have shown increasing interest in using virtual patients to improve empathy.	Medical students were split into 3 groups with each group interacting with a VP portraying a specific scenario. Each group then interacted with a SP portraying the same scenario. Students were assessed for their responses during each interaction.	1) Students who were given feedback about their empathic responses during their VP interaction elicited more empathic opportunities.<br>2) Students given empathy-feedback had overall higher rated empathy<br>3) Students in different groups displayed different levels of empathic response when faced with different empathic opportunities.<br>4) Students in the control group were rated lower in terms of building relationships with the patient.	Providing immediate feedback on empathy after a simulated interaction is associated with overall increased empathy in both VP and SP interactions<br>The results show that VPs offer significant advantages over SPs and are an acceptable and effective modality for increasing empathy.

Fragkos & Crampton	2019	The Effectiveness of Teaching Clinical Empathy to Medical Students: A Systematic Review and Meta-Analysis of Randomized Controlled Trials	Clinical empathy is important to patient care but is greatly complex. There have been many studies on various empathy interventions, but they do have their limitations. This study aims to examine if empathy interventions in medical students are effective and how confounding factors potentially moderate this effect.	This is a systematic review and meta-analysis using PRISMA.<br>A search was done from January 1 1947 to January 31 2018 for randomized controlled studies on empathy interventions in medical students.	16 studies were included.<br>1) There is a moderately positive effect of students developing empathy after an intervention compared to that of a control group. The effect size was steadily above 0.55-0.60.<br>2) Age, country, year of study have an impact on the effectiveness. Europe and Year 2 students seemed to have a larger impact.<br>3) Narrow scope empathy measures had a lower effect than broad-scope measures.<br>4) Mixed interventions had the largest impact on effectiveness, while didactic interventions were not significant.	Broader empathy measurements may be useful but they exhibit significant heterogeneity.<br>Self-reported instruments had a larger effect size possibly due to social desirability effect, possibly more so among women.<br>A more consistent and less heterogenous instrument is needed.<br>Empathy interventions should include mixed methods and rehearsals, target students towards the end of medical school, assessed objectively by experts or SP and the definition of empathy should be broad.
Ghetti et al.	2009	Burnout, psychological skills, and empathy: Balint training in obstetrics and gynaecology residents	Balint group training is currently used in over 50% of family medicine residency training programs. Balint<br>sessions not only provide training and skills in understanding the doctor-patient relationship and help develop empathy, but they may also be a resource for improving job satisfaction and reducing physician burnout.	36 OBGYN residents participated in a monthly 1h Balint group for a year.<br>This is a pretest-posttest quantitative study utilising the Psychological Medicine Inventory, the Maslach Burnout Inventory, and the Jefferson Scale of Physician Empathy.	1) Psychological Medicine Inventory overall scores improved at 12 months when compared with baseline scores.<br>2) Jefferson Scale of Physician Empathy scores remained unchanged at 12 months<br>3) At 12 months, the number of residents with high burnout scores in each subscale decreased.	Our study showed improvement in some aspects of behavioural-medicine skills of obstetrics and gynaecology residents at the 12-month assessment. Residents reported an improvement in ability to use consultation, ability to make appropriate treatment decisions based on patients' psychological needs, and ability to be psychologically therapeutic with patients. Our study found no difference in baseline and 12-month Jefferson Scale of Physician Empathy scores.

Gibon et al.	2013	Is it possible to improve radiotherapy team members' communication skills? A randomized study assessing the efficacy of a 38-h communication skills training program	Good communication with patients and among team members is essential in optimizing patient care.	A randomized control trial was conducted on 80 members of radiotherapy teams (51 intervention, 29 control). The intervention consisted of a 38h communication skills training programme using modalities such as didactic lectures and roleplays. Assessment of participants consisted of a qualitative analysis of a SP interview and a self-reported questionnaire on competency in communication skills. Assessments were conducted before and 4 months after the intervention was completed.	1) Intervention group showed a significant increase in team-resource-oriented turns of speech compared to control group<br>2) Intervention group showed a significant increase in the rate of questions asking compared to control<br>3) Intervention group showed a significant increase in empathy and negotiation compared to control<br>4) Intervention group showed a significant increase in emotional words compared to control<br>5) Intervention group reported increased confidence in ability to communicate with SP about her concerns and with colleagues	This study shows a communications skills training programme is feasible and effective at improving communication in radiotherapy teams, with participants showing improvement in their assessment, informative, and supportive skills. This programme is also effective at improving patient-centredness in team members. Role playing in groups can contribute to this improvement but trainer feedback needs to be adjusted to the individual skill level of the participants.
Gozalo et al.	2019	Application of a mindfulness program among healthcare professionals in an intensive care unit: Effect on burnout, empathy and self-compassion	Professional wear or ''burnout syndrome'' is a normal but undesirable reaction to chronic interpersonal and emotional stress in the working environment. Training in ''mindfulness'' as a personal wellbeing tool that has demonstrated its efficacy in the clinical setting, is seen as a means by which to achieve this	8 physicians, 13 nurses and 11 nursing assistants participated in 8 weekly mindfulness practice (5-8min).<br>This is a pretest-posttest quantitative study utilising the Jefferson Empathy Scale, the Maslach Burnout Inventory----Human Service Survey, the Five Facets of Mindfulness	1) The Jefferson Empathy Scale showed no significant global changes as a result of the program.<br>2) The global scores of the Self-Compassion Scale were seen to increase significantly as a result of the intervention.<br>3) FFMQ showed no global pre-post program modification. However, the scores corresponding to	A brief and personal practice-based mindfulness training program supported by the creation of a WhatsApp virtual community group among healthcare demonstrated psychological and cognitive effects that may afford wellbeing and have an impact upon burnout by reducing emotional exhaustion and incrementing self-compassion. All this in

			objective in the workplace. In general terms, it may be affirmed that programs based on mindfulness result in modifications in participant attitude toward personal thoughts, sensations and emotions, as psychological changes that appear to have neuroanatomical correlates. In the healthcare setting, the application of such programs would extend beyond the individual context of the professional, strengthening skills that are crucial for the therapeutic relationship.	Questionnaire, and the Self-Compassion Scale.	‘‘Observation’’ and ‘‘Non-reactivity to inner experience’’ were seen to increase, while those corresponding to ‘‘Acting with awareness’’ and ‘‘Non-judging of inner experience’’ decreased.<br>4) The MBI dimension "emotional exhaustion" showed positive global effect, while for the dimension "personal achievement", the positive effect only reached statistical significance among the for physicians.	turn may result in improved quality patient care.
Graham et al.	2014	A patient-led educational program on Tourette Syndrome: impact and implications for patient-centered medical education	Being a patient-centred practitioner requires increased practitioner empathy, which ultimately leads to more effective medical practice. Recent literature indicates that physician empathy increases patient comfort in reporting symptoms, patient satisfaction and patient adherence to physician recommendations.<br>Studies using the JSE show a decline in empathy throughout medical education, decreasing as soon as the 1st year, with overall empathy higher in	79 medical residents participated in a 1h patient-led presentation by one or two teenagers diagnosed with Tourette Syndrome.<br>This is a pretest-posttest mixed method study utilising the primary 10-item subscale of the JSE (perspective-taking) and participant comments were elicited via an open-ended prompt on the postpresentation assessment.	1) Jefferson Scale of Physician Empathy (JSPE) perspective-taking primary subscale revealed a significant increase from total pre-presentation scores to total post-presentation scores, indicating that participants endorsed a more empathic view following the patient-led presentation<br>2) Four themes identified from participant responses include: Medical Knowledge of Tourette Syndrome, Empathy for Patients, Supportive Resources for Patients, and	Patient-centred physician education may provide a significant and useful supplement to traditional medical education. An increase in physician empathy following a patient-led presentation may have a positive impact on patient care. The authors report that previous experience with use of the same format with other conditions (eg. breast cancer) suggests the benefit of this format across various medical conditions.

			earlier, preclinical years as compared to later clinical training years. A variety of methods have been utilised to increase physician empathy, but less commonly used are educational programs in which patients play a leading role.		Elements of Humanistic Medicine.	
Graham et al.	2016	Medical Humanities Coursework Is Associated with Greater Measured Empathy in Medical Students	Empathy is an intrinsic professional value, promotes better clinical outcomes and prevents burnout. However, it is known to decline during medical education and patients have found it to be lacking in physicians. Medical humanities have been suggested to empathy.	Reading literary fiction has been suggested to enhance "theory of mind", which can develop empathy clinical relationships. 25 medical school students were enrolled in an elective medical humanities coursework. They were assessed with the JSE-S at the start and at the end of the academic year, and their results were compared to that of 43 control group students.	56% of the experimental group students had a statistically significant increase in JSE scores as compared to 28% of the control group. 44% of the experimental group students had a decrease/failed to grow in JSE scores as compared to 72% of the control group.	Humanities coursework may directly cultivate empathic reasoning and cognitive skills, which are neglected in the standard medical school curricular. The interpersonal exposure of students to faculty physicians who valued the humanities may have contributed to the improvement in empathy.
Han & Pappas	2018	A Review of Empathy, Its Importance, and Its Teaching in Surgical Training	There is extensive literature supporting the fact that doctors who have and demonstrate empathy deliver better care, and a physician who provides compassionate care is more effective than one who does not. Empathy, appears to have a significant positive and indirect effect on long-term patient satisfaction and	This is a review of empathy, it's importance and some methods of teaching empathy in surgical training. Two studies highlighted included a standardised patient video series for breaking bad news and a high stress simulation with faculty mentoring	The standardised patient series presurvey and postsurvey results showed statistically significant increased self-reported competence for “asking open-ended questions,” “fire warning shot,” “obtaining informed consent,” and “delivering a disappointing outcome.” The high stress simulation	That surgeons may be uniquely susceptible to the loss of empathy during training is problematic and bears addressing. Empathy is a skill that can be taught, in the form of didactics, standardised patient simulations and role-playing, and surgery apprenticeship.

			health outcomes in terms of self-perceived health status and post-operative satisfaction with their surgeons. It is likely for surgeons, a certain degree of suppression, if not detachment, may be necessary in order to remain effective in the face of suffering (inflicting pain to treat the patient).		with faculty mentoring found variable increases in emotional quotient inventory scores in the majority of residents, with corresponding increase in patient satisfaction measures.	
Harlak et al.	2008	Communication skills training: effects on attitudes toward communication skills and empathic tendency	Many medical educators and physicians appreciate the importance of communication skills (CS) for clinical practice. The long-term effects of CS training have not been assessed yet; however, studies have shown that students hold positive attitudes toward CS. Physician’s empathic skills are critical for doctor-patient interaction.	59 first-year medical students participated in a 30h communication skills training.<br>This is a pretest-posttest quantitative study utilising the Communication Skills Attitude Scale, and the Empathic Tendency Scale.	1) There was moderation in attitudes and empathic tendencies after the programme.<br>2) Comparisons of empathic tendencies between pre- and post-tests indicated that in low empathy group, training increased students’ empathic tendencies whereas in high empathy group it did not.<br>2) Comparisons of attitude towards CA between pre- and post-tests indicated that in positive attitude group, training decreased students’ attitude towards communication skills whereas in negative attitude group it did not.	In view of the authors' findings, their curriculum is in need for further examination and modification in order to create positive changes in students' attitude towards communication skills.
Hart et al.	2006	Enhancing parent–provider communication in ambulatory paediatric practice	Effective communication skills has been shown to produce better patient outcomes. Yet, studies have shown that parent-provider	28 paediatric residents took part in a intervention to enhance interpersonal communication skills with families they treated.	The total amount of interpersonal communication utterances as measured by RIAS and the amount of	A brief, resident-focused intervention can be effective in improving the communication skills of residents. This improvement

			communication within primary care may not be optimal. Previous studies investigating communication skills training in paediatrics were limited by a narrow focus or had no evidence of outcomes in the clinical setting.	Audiotaped interviews in the paediatric clinic before and after the intervention were analysed using the RIAS coding system. Patient perceptions of the consultation were scored using the P3C measure and the P-MISS measure.	empathy/reassurance displayed by the residents increased over time. No change in the parent's perception of the residents were noted. Parent satisfaction with the care received also increased.	may result in increased parent satisfaction with care.
He et al.	2019	The art of observation: a qualitative analysis of medical students' experiences	Arts in medical school curricular has potential in improving observation skills and promote reflection. Arts-based courses have also shown to help students develop professionalism and empathy, which are basic competencies in medical education.	65 Years, 1, 2 and 3 medical students participated in an art-based elective comprising of 7 two-hour sessions. Students wrote course evaluations reflecting on their experience of the elective and described their perceptions, thoughts and feelings. This qualitative study used grounded theory to analyse the 65 evaluations.	Three major themes were identified:<br>1) enhanced observation skills<br>2) improved physician socialization, development of personal and professional identities, tolerance of ambiguity, development of empathy and compassion, reconstruction of the patient narrative<br>3) decrease in burnout	Students found the small group discussion useful in building teamwork, a major focus in healthcare. Medicine and art have several parallels, and art can be applied to promote observation skills, a humanistic view, physician socialization and physician well-being.
Hojat et al.	2013	Enhancing and sustaining empathy in medical students	Empathy is important to the physician-patient relationship, part of clinical competence ratings in medical school, and promotes positive patient outcomes. Studies on interventions to promote empathy which have been successful were limited by a small sample size, a lack of control group or long-term follow-up. Hence, this paper	All 248 students were first administered the JSE. In Phase 1, 129 students in the experimental group watched 22 video clips from 3 movies (The Doctor, First Do No Harm and Wit) for one hour. All students were administered the JSE again after Phase 1. Ten weeks later, in Phase	Phase 1:<br>1) There was a statistically significant increase of 2.2 points in the empathy scores of students in the experimental group, but no change was noticed in the control group.<br>2) Men improved by 2.7 points, which was more than that of women (1.6 points).<br>Phase 2:<br>1) The reinforced group had	The authors noted it was encouraging that this study supports that empathy can be enhanced and sustained by medical students after targeted interventions, since empathy has been reported to decline during undergraduate and graduate medical education.<br>Considering this study's findings, and recent studies linking physician empathy

			aimed to study if 1) medical students' empathy would be enhanced by watching and discussing video clips of medical encounters from certain selected movies 2) enhanced empathy can be sustained when reinforced by supplementary educational activities	2, the experiment group was randomly divided into two groups and one group attended a lecture and slide presentation about the importance of empathy in patient care (reinforcement group), while the other (not reinforced) and the control group watched a movie, Something the Lord Made. All students were administered the JSE-S again.	sustained their improvement in empathy scores, but not in the non-reinforced group.	and better patient outcomes, the authors argue that leaders in academic medical centres should work on an action plan to design and implement programs to enhance and sustain in medical students and physicians.
Holton-Burke et al.	2019	The addition of virtual reality into the neurology curriculum	Virtual reality can overcome challenges of cost, safety or setting and play a role in future education of healthcare professionals. They can augment existing tools, ensure a uniform training, broaden clinical exposure and develop empathy.	This abstract briefly describes a VR intervention and educational video on epilepsy for Year 3 medical students.	There was a significant increase in competency of treating epilepsy. There was also an increase in empathy and better communication with patients with seizures.	VR is easy to implement and well received by learners. This emerging technological tool has the potential to add-on to other teaching methodologies.
Johnsen & Lok	2008	An evaluation of immersive displays for virtual human experiences C3 - Proceedings - IEEE Virtual Reality	Virtual human experiences are gaining in popularity and there is growing interest in integrating them in the medical curriculum.	27 medical students participated in a study comparing 2 types of displays for a virtual patient experience. Students completed self-assessments prior to the intervention and were assessed on their interaction with the virtual patient.	1) Students reported that the head mounted display was more immersive 2) The head mounted display made the appearance of the virtual patient fit the role less 3) Students who used the head mounted display rated their empathy higher 4) No significant change in behaviour was seen in both groups of students	The novelty and encumbering nature of using VR experiences drew attention away from the VP and may have caused inaccurate self-assessments of student empathy. However, this intervention was well received and should be considered as a cost-effective way of training students.

					5) Students showed different responses to the VP sneezing<br>6) No difference was observed in the grades given for the treatment plan and diagnosis of the patient	
Johnson et al.	2013	Does communication skills training make a difference to patients' experiences of consultations in oncology and palliative care services?	Effective communication is important in the doctor-patient relationship. Current literature evaluating communication skills training has largely neglected the patient's perspective. The expression of empathy is also important and has been one of the main aims for communication skills trainings.	21 HCPs (12 intervention, 9 control; 9 doctors intervention, 8 doctors control) collected evaluation from their consultations with patients, with the patients rating their doctors using the CARE measure. A supplementary questionnaire to find out about the patient's satisfaction was also given. The intervention group took part in 3-day communication skill training course. Pre-post intervention data collection was done.	1) No significant difference was noted in the CARE measure before and after the training in both the intervention and control group.<br>2) More patients reported that they were satisfied with the length of time of the consultation and that their doctor understood them more in the control group. No change was seen in the intervention group<br>3) A slight trend towards improvement in CARE was seen in the intervention group for those with low scores at baseline with a trend in the opposite direction for those with high scores at baseline.	While the intervention was not successful at increasing empathy levels for participants who did not have low baseline scores, the feedback HCPs obtained were well received.
Junek et al.	1979	Teaching interviewing skills by encountering patients	Previous studies have shown that despite the importance of interviewing skills in a doctor-patient relationship, 1st year psychiatry residents lack many of these skills.	6 first year psychiatry residents took part in a 12 week intervention focusing on interview skills. Residents were assessed using a interview with a patient before and after the intervention. They were	The residents improved at the end of the intervention	Participants found that group discussion was useful and was more effective than the patient interviews. Good role modelling by supervisors was valued by the participants.

				assessed using the Truax scales and Barrett-lenard relationship inventory.		
Kane et al.	2018	The effects of a longitudinal patient experience on the enhancement of empathy in first and second year medical students	Empathy is an essential component of patient care with benefits for both the patient and physician. The Longitudinal Educational Experience Advancing Patient Partnerships programme was previously piloted in Weill Cornell Medical College.	207 medical students took part in a 4-year longitudinal patient experience programme during their time in school. Students completed a JSPE before and 1 year into the programme. The CARE measure was completed by the patients 2 years after the start of the programme to evaluate the students.	1) No significant changes in empathy scores were noted. 2) The patients had a very positive impression of the students based on the CARE questionnaire	While the traditional medical curriculum teaches about empathy, longitudinal patient experiences provides students to practice behavioural empathy by engaging students with ongoing interactions with patients.
Kaplan-Liss et al.	2018	Teaching Medical Students to Communicate with Empathy and Clarity Using Improvisation	Many adverse patient outcomes are caused by poor clinician-patient communication. Hence, medical schools started communication training. Empathy is an essential learning objective and can influence patient satisfaction, clinical outcomes, and professional satisfaction. Thus, a communication skills training program incorporating improvisation was developed to improve medical students' empathy.	76 students participated in the Communicating Science elective where improvisation techniques were taught over six weekly three-hour sessions. There were interactive lectures, discussions, exercises, role-playing, and on-camera interviews.<br>A course evaluation was conducted by the students after the intervention	96% students gave the course the highest rating. Students expressed that the techniques were relevant to their future career and reinforced their learning from OSCEs. All of them expressed they wanted more the course content to be incorporated in the school curriculum.<br>Students felt that also commented that they were able to better understand a patient's<br>perspective, explain health issues using less jargon, and listen more intently. Some even stated it helped them learn medical material more easily.	Teaching in teams of two was more effective and the course content was useful for interprofessional education.<br>It is difficult to find time and appropriate faculty for teach communication skills. It is important to embed such training throughout medical school, into residency and train faculty members to role model, teach and assess communication skills.

Karaoglu & Seker	2011	Looking for winds of change with a PBL scenario about communication and empathy	The empathic response is essential for a good communication and should include effectiveness in eliciting information about patient beliefs and feelings and conveying respect and caring. Although medical students enter medical school with a desire to care for people in need, due to the challenges they faced during their clinical training they become less empathic toward their patients. Consequently, major task of medical educators which is to help maintain and increase empathy for patient becomes ineffective. It is important for entry-level students to draw connections between the communication skills and performance quality in clinical practice.	282 first-year medical students participated in three sessions of three scenarios of communications problem-based learning. This is a pretest-posttest quantitative study utilising the Communication Skills Attitude Scale, the Empathic Tendency Scale, and the Empathic Skill Scale.	1) Although there was a significant increase in the positive attitude scores of the all group (p=0.00), negative score change was not significant (p=0.16).<br>2) There were a non-significant increase in empathic tendency scores<br>3) There were a non-significant increase in empathic skills scores	This problem-based learning scenario had significant positive effects on positive attitude scores, while there was also non-significant increase in empathic tendency and empathic skill.
Kataoka et al.	2019	Can communication skills training improve empathy? A six-year longitudinal study of medical students in Japan	Empathy has been described as an important component of overall physician competence. Empirical research has shown that medical student's empathy is correlated with faculty's ratings of clinical competence), and physician empathy is significantly associated with positive patient outcomes in diabetic	116 medical students participated in a 4h communication skills training workshop, with 69 complete responses. This is a pretest-posttest longitudinal quantitative study utilising the Jefferson Scale of Empathy	Jefferson Scale of Empathy (JSE) Score increased significantly immediately after the completion of the program but was not sustained for a longer time.	Empathy can be taught to medical students by participation in communication training programs. Targeted educational programs may not exert a sustained effect, suggesting that programs to enhance empathy may need to be reinforced frequently during medical training to

			patients. Research suggests that empathy in medical students can be enhanced by participation in targeted educational programs.			maintain their long-term effects.
Kelm et al.	2014	Interventions to cultivate physician empathy: a systematic review	Despite considerable evidence demonstrating the benefits of physician empathy for patients and physicians, empathy is at a lower than ideal level in medicine. Studies indicate that physicians often overlook or miss empathic opportunities during patient encounters, and tend to spend significantly more time and energy on biomedical inquiry and offering medical explanations to patients. The lack of empathy among physicians and the decline in empathy throughout medical training offer reasons for concern, especially given the relationship between physician empathy and patient health and well-being. It is incumbent upon medical educators, and the field in general, to investigate methods to enhance medical student and physician empathy.	A search for studies utilising the following search terms: 'empathy or caring or compassion', 'medical students or physicians', 'medical education or clinical competence or training or workshop', 'communication', and 'doctor patient relation or interpersonal communication' was conducted using PubMed, EMBASE, Web of Science, and PsychINFO. 64 English language quantitative studies that assessed changes in empathy due to interventions among medical students, residents, fellows, and physicians were identified.	42 (66%) of the 64 reviewed studies reported a statistically significant increase in empathy. 14 studies (22%) showed no significant change in empathy. Finally, eight studies (12%) were classified as “mixed” because they reported some measure with no significant change in empathy and another measure with a significant increase in empathy.	Targeted interventions may be able to cultivate physician empathy.
Kleinsmith et al.	2015	Understanding Empathy Training with Virtual Patients	The use of virtual characters in medical education is becoming more and more	110 third year medical students interviewed VPs and SPs. Their responses	Medical students responded more empathetically to VPs than SPs/	More work needs to be done to understand the implementation of VPs in

			commonplace. Empathy has been shown to improve patient outcomes.	were rated using the ECCS. Self-reported surveys were used to collect the student's perception of the programme and JSPE was administered as well.		medical education as students respond more empathetically to VPs based on the results. Furthermore, the other known benefits VPs have over SPs make it a useful tool in medical education.
Koblar et al.	2018	Developing Empathy: Does Experience Through Simulation Improve Medical-Student Empathy?	Empathy is valued as an important personal quality to effectively practice medicine. The ability to effectively and empathetically communicate with both patients and their relatives, and with other professionals, is defined as a fundamental outcome of the University of Adelaide's undergraduate Bachelor of Medicine and Bachelor of Surgery Graduate Program. However, despite extensive evidence demonstrating the multiple benefits of physician empathy for patients and physicians, it has been demonstrated that empathy is at a lower than ideal level in medical professionals. Studies indicate that doctors often overlook or miss empathetic opportunities during encounters with patients	62 fourth year and fifth-year medical students participated in a stroke simulation, with half acting as patients and half acting as caregivers. This is a pretest-posttest mixed methods study utilising the Jefferson Scale of Physician Empathy and participants' reflective writing.	1) JSPE scores increased for all participants (patients & carers), with no statistically significant difference with regard to mean changes in JSPE scores between patients and carers.<br>2) Overall, it is clear the most emphatic responses related to increasing understanding and insight of what it is like to receive care as a patient with a disability and identifying with the difficulties faced on a daily basis, with the most common responses being recognition of the difficulties in time taken to complete tasks, receiving 'odd looks and stares,' reporting an 'eye-opening or valuable experience' a myriad of physical symptoms, feeling judged, and insight into understanding stigma and the experiences of disabled patients.	An experiential learning role-play, in which students undertake a simulated experience as either a stroke patient or a caregiver, is effective in improving medical-student empathy.
Kommalage	2011	Using videos to introduce clinical	Interpersonal relationships are important in improving	2 videos on thalassaemia and haemophilia were	JSPE scores increased after the intervention for both	Video presentation of clinical material can be useful to

		material: Effects on empathy	patient outcomes. Previous studies have suggested that early exposure to clinical material can help to develop empathy in students.	shown to preclinical students and discussion was encouraged. JSPE was administered before and after the intervention	male and female students although female students had greater mean change in JSPE scores	increase empathy in a traditional medical school.
Kramer et al.	1989	Increasing empathy among medical students	Empathy and communication skills are essential components of patient care with benefits for both the patient and physician. Studies have shown that communication skills can be learnt but are also easily forgotten, with the introduction of technology in medical practice being a major barrier to effective communication.	40 fifth year medical students and their 10 tutors took part in this observational study. The students were split into 4 groups to compare the effectiveness of allowing tutors to impart skills to students with teaching the students directly in workshops. Students were observed a week before, 1 week after, 6 months after, and 12 months after the workshop to assess their communication skills in a medical interview.	1) All participants who actively participated in the interpersonal skills workshops showed significant and lasting increase over time in "net supporting" behaviour.	Contrary to previous studies that suggest behaviours and attitudes can be transmitted through role models, the results of this study suggests that a direct teaching approach is more effective at developing and maintaining empathy.
Krasner et al.	2009	Association of an educational program in mindful communication with burnout, empathy, and attitudes among primary care physicians	There has been a major decrease in the percentage of graduates entering careers in primary care in the last 20 years, with reasons related to burnout and poor quality of life. Even though the problem of burnout in physicians has been recognized for years, there have been few programs targeting burnout before it leads to personal or professional impairment and very little data exist about	70 primary care physicians participated in an 8 week (24.5h) intensive course which included mindfulness meditation, self-awareness exercise, narratives about meaningful clinical experience, appreciative interviews, didactic material, and discussion, which was followed by a 10 month (25h) maintenance phase.	1) JSPE scores improved, with standing in the patient's shoes and perspective taking demonstrating significant positive changes.<br>2) Mindfulness showed the largest effect sizes and positive change.<br>3) Maslach Burnout Index showed improvement over all 3 subscales.<br>4) Physician Belief Scale improved significantly.<br>5) The profile of Mood States showed moderate	Physicians who participated in a CME program on mindful communication showed improvements in measures of well-being and demonstrated an enhancement in personal characteristics associated with a more patient-cantered orientation to clinical care.

			their effectiveness. One proposed approach to addressing loss of meaning and lack of control in practice life is developing greater mindfulness —the quality of being fully present and attentive in the moment during everyday activities.	This is a pretest-posttest quantitative study utilising the Jefferson Scale of Physician Empathy, the Maslach Burnout Inventory, the 2-Factor Mindfulness Scale, the Physician Belief Scale, the Mini-markers of the Big Five Factor Structure personality scale, the Profile of Mood States.	effect sizes in the total score.<br>6) For most measures, similar degrees of improvement were seen after the 8 week intensive intervention, at the conclusion of the monthly maintenance phase, and 3 months beyond completion of the program. Several short-term improvements did not persist (physician empathy: compassionate care; profile of mood states: tension and confusion; and personality factors: extraversion, agreeableness, and openness) although 5 improvements developed over the long term that were not apparent at 8 weeks (burnout: depersonalization; profile of mood states: depression and fatigue; and personality: conscientiousness and emotional stability).	
Kushner et al.	2014	An obesity educational intervention for medical students addressing weight bias and communication skills using standardized patients	A major challenge facing medical educators today is to adequately train current and future physicians in the prevention and treatment of chronic illnesses. Two topics in obesity education that particularly merit increased attention are stigma and communication	127 first-year medical students participated in a 1h roleplay with an overweight or obese simulated patient after some readings.<br>This is a pretest-posttest quantitative study utilising a self-created 16-item questionnaire	1) Significant improvement between baseline and the immediate follow-up survey was observed in all three scales, ranging from small improvement in stereotyping and empathy, and a much larger mean improvement in confidence.<br>2) At one year, negative	Medical student attitudes, beliefs and comfort in communication skills regarding obesity can be improved after a structured educational intervention that included a SP encounter.

			skills. Patients with obesity are also a common target of derogatory humour by students, attendings and residents. It can be hypothesized that tackling these attitudes and skills regarding obesity during undergraduate education may increase the preparedness of physicians to provide more frequent and empathetic obesity care.	including components on Negative Obesity Stereotypes, Empathy for Obese Patients, Confidence in Clinical Interaction with Obese Patients	obesity stereotyping had regressed to baseline levels and the modest decrease in stereotyping at the immediate follow-up survey had disappeared. However, gains were maintained for the mean empathy and counselling scale scores which remained statistically significant from baseline.	
Lim et al.	2011	Being-in-role: A teaching innovation to enhance empathic communication skills in medical students	The communication of empathy is considered a key element in the success of clinician–patient interactions. An empathic doctor–patient relationship is important in developing a therapeutic alliance and increases the patient's motivation to actively participate in treatment. Alarmingly, there is a growing public perception that clinicians might have become too ‘‘detached’’ to care, and this perception has been further reinforced by cross-sectional and longitudinal studies on the decline in the manifestation of empathy among medical students	77 fifth-year medical students participated in an 1h acting workshop, with another 72 students participating as a control group.<br>This is a pretest-posttest quantitative study with a control group that utilised the Jefferson Scale of Physician Empathy, and observed structured clinical examinations that were evaluated using the Behaviour Change Counselling Index (BECCI)	1) Students in the intervention group reported significantly higher JSPE scores as compared to students in the control group after intervention.<br>2) BECCI scores were higher in the intervention group than students in the control group, indicating higher competence in consultations about behaviour change.<br>3) Tutors rated students in the intervention group as having significantly better OSCE performance than students in the control group.	Just 1h of the actor-facilitated teaching innovation 'How to act-in-role' was effective not only in increasing medical students' empathy but also their competence in consultations about behaviour change, indexed by BECCI scores, as well as the students' OSCE performance.
Liu et al.	2016	Reflective Writing for Medical Students on the	Studies have shown that empathy levels among surgeons are lower than	Medical students in their surgical clerkship took part in a reflective writing	1) Students reported that they found the workshop worthwhile and wanted	A reflective writing workshop is not only effective in promoting empathy but also

		Surgical Clerkship: Oxymoron or Antidote?	other specialties. While reflective writing has been shown to be effective in curbing the decline of empathy, it has not been attempted in the surgical setting.	workshop. A survey was administered 4 weeks before the start of the workshop and immediately after the workshop.	more opportunities for reflective writing.<br>2) Self-reported empathy decreased after the workshop<br>3) Students generally had a positive experience with the workshop	acts to increase receptivity towards more of such workshops. It also acts to improve student perception of the surgical clerkship, thus showing there is room for reflection even in surgery. Observing peers reflect on their emphatic experiences with patients may lead to a deflation of self-rated empathy.
LoSasso et al.	2017	Enhancing Student Empathetic Engagement, History-Taking, and Communication Skills During Electronic Medical Record Use in Patient Care	There is an increase in use of electronic medical records (EMRs) and history taking using computers in clinical practice. It has been reported that physician-patient communication and physicians' empathic engagement may be adversely affected by EMRs.<br>To tackle this challenge, the authors seek to examine whether a specific intervention on proper use of EMRs) in patient care could help improve medical students' empathic engagement, and to test the hypothesis that the training would reduce communication hurdles in clinical encounters.	All 70 participants attended a regular clinical training on EMR. 38 Year 3 medical students in the intervention group attended another one-hour training on EMR-specific communication. The intervention was based on the SALTED mnemonic and included a discussion and 4 role-plays<br>All participants had a video-recorded SP encounter post-test and assessed using a communication skills and history-taking skills Likert rating scale by the faculty and SPs. They also rated the students with the JSPPPE. All participants were also	1) The JSE mean score for the intervention group increased while that of the control group decreased, but these changes were not statistically significant. The post-test mean JSE score of the intervention group was higher than the control group, though the difference was not statistically significant.<br>2) Faculty rating: The intervention group was rated significantly higher on the JSPPPE, communication skills and history-taking skills than the control group. Effect size estimates indicated that this difference was of practical (clinical importance)<br>3) SP rating: The intervention group was rated higher on history-taking skills and JSPPPE, but only	1) This study built on existing studies on the impact of the SALTED training. The SALTED technique lays an important foundation for empathic engagement in patient care<br>2) The role plays were expected and found to be improve empathic engagement.<br>3) This study emphasises the importance of including EMR-specific training in the medical school curriculum.

				administered the JSE pre-test and post-test.	the former was of statistical significance.<br>4) The faculty mean ratings on all three assessments were significantly higher than that of the SP.	
McDonald et al.	2015	Clinical realism: a new literary genre and a potential tool for encouraging empathy in medical students	We live in a post-modern society where a technocratic approach to medicine is no longer considered enough or desirable. Twenty-first century patients are looking for person-centred care: they want to be listened to and to have a dialogue with their doctor, to be healed rather than cured. For the purposes of this course, one of the authors (PM) created a new genre, clinical realism. This is defined as "Fictional writing where health problems are systematically represented, not as a metaphor, not as a plot point, and not as the central topic of the writing, but as a part of a character's personal identity and day to -day experience".	6 third-year medical students participated in 4 weekly workshops on narrative medicine.<br>This is a qualitative study based on the work produced by the students, analysed thematically using a constructivist approach to meaning making.	The quality of writing produced by students on the Student Selected Component (SSC) was high, and their writing was often very moving. Analysis of the writing showed that the students were able to produce successful portrayals of the day- to-day experience of living with a chronic disorder.<br>As well as demonstrating cognitive empathy, the ability to understand how another person feels, there was also some evidence of affective empathy, experiencing the emotions that other people are feeling.<br>A recurrent theme was that during the process of creating and then repeatedly writing about the same fictional character the students felt that they developed a deeper empathic understanding of their character, even when they initially felt little connection with them.	A medical realism approach to creative writing, combined with creating and then repeatedly writing about the same fictional character, offers the potential for a low-cost intervention in a safe and convenient environment, which can potentially help to increase the empathic ability of students.

McManus et al.	2019	Establishing and evaluating a Balint group for fourth-year medical students at an Irish University	Empathy has been found to decline in medical students as they reach their clinical years. Medical teachers note that students who are unable to examine and accept their own psychological lives find it difficult to connect empathically with others. Hence, developing reflective capacity is important, and this can be achieved using a Balint group.<br>This study aims to establish and evaluate the impact of a 6-week Balint group on empathy and resilience in fourth-year medical students during their psychiatry rotation.	28 Year 4 medical students participated in the intervention. Each Balint group met weekly for 1 hour over 6 weeks. This is a pretest-posttest mixed method study utilising the JSE-S, the Brief Resilience Scale (BRS) and focus groups after the intervention.	1) There was a significant improvement in mean empathy score from 109 to 116 on the JSE-S.<br>2) There was an improve in mean resilience score on the BRS from 3.77 to 3.98, though not statistically significant.<br>3) Themes discussed at the focus groups were:<br>- A new understanding of reflective practices<br>- New insights into case dynamics<br>- Negative role models<br>- Feelings towards the patient<br>- An increased sense of professional identity<br>- A shared sense of belonging with other students	This intervention was unique as participants could reflect and discuss on their own experiences and brought a 'realness' to the Balint groups. Despite a lack of correlation between Balint groups and resilience, this study mirrors findings from previous studies and provides evidence to the efficacy of Balint groups to promote empathy amongst medical students.
Misra-Hebert et al.	2012	Improving empathy of physicians through guided reflective writing	Effective communication skills and greater physician empathy has been shown to produce better patient outcomes. Furthermore, both can be trained, with narrative writing being one of the more effective modalities.	20 physicians took part in a reflective writing training workshop while 10 more only received the materials for the workshops and another 10 did not receive the materials or the training. All participants completed the JSPE at the start, middle, and end of the intervention. Qualitative analysis of the reflective writing pieces was also used.	1) About half of the reflective writing pieces showed detached concern while the other half showed compassionate solidarity with the situation.<br>2) A majority of reflective writing pieces explored negative rather than positive emotions.<br>3) The intervention group showed an improvement in JSE score	The reflective writing practice was positively received, and participants appreciated that it was conducted during protected time so that they could focus on reflection. The use of peer group discussions can allow for sharing and processing of negative experiences and may lead to greater empathic understanding.

Moorhead & Winefield	1991	Teaching counselling skills to fourth-year medical students: a dilemma concerning goals	Counselling and communication skills have become more common in medical training.	63 fourth year medical students completed the Empathy Rating Scale once at the start of their General Practice attachment and once at the end. During the attachment, students were trained on communication skills on top of the usual clinical curriculum.	1) No statistically significant increase in empathy scores were noted<br>2) The most common response to empathetic trigger statements was to give advice	The short 15hrs of instruction the students received during their attachment was likely not enough to improve their empathy, thus length of intervention is important.<br>Different degrees of and methods of expressing patient-centredness may well be relevant for different presenting problems.<br>There is a growing resistance to adopting patient-centred communication among medical students, and providing clearer evidence on the benefits of this might be useful to overcome this resistance.
Muszkat et al.	2010	Teaching empathy through poetry: a clinically based model	Medical humanities can increase medical student's empathy. However, they have been criticized as being not clinically relevant, insufficient doctor participation and role-modelling. To overcome these challenges and the dichotomy between their emotional and intellectual competencies, a clinically based medical humanities model was developed.	122 medical students participated a clinically based medical humanities programme.<br>They evaluated the programme with a survey on a 7-point Likert scale.	Students rated their empathy (understanding of what it's like to be a patient) a mean of 5.70.<br>Students favoured clinical facilitation and obligatory student participation.	The model which is clinically relevant in content, timing and facilitation has been perceived by students to be effective in increasing empathy.
Noordman et al.	2019	Training residents in patient-centred communication and empathy: evaluation from patients,	Patient cantered communication and empathic communication have been shown to improve patient outcomes and	9 residents from various specialties took part in a 3-day training programme focused on patient centred	1) After training, patients more often received what they expected from the consultation<br>2) Statistically insignificant	1) Training in patient centred communication can halt the decline in empathy<br>2) Most patients consider affect-ordered communication

		observers and residents	physician well-being. However, there is a downward trend in physician communication skills and medical student empathy.	communication and empathy. Pre-test evaluation of self-recorded consultations was done using the MAAS-global scale. Patients rated the residents pre and post-test using the QUOTE COMM scale, STAI, CARE. Observers of the consultations evaluated the residents using a self-developed score and the MAAS-Global.	slight downward trend in anxiety levels of patients after intervention<br>3) residents showed significant improvement on patient ratings of physician empathy via CARE<br>4) Residents' communication skills did not change significantly after the training as rated by observer<br>5) Insignificant decrease in number of consultations which started with the resident setting agenda after the training<br>6) Intrusion of computer use improved significantly	aspects the most important and want a frank doctor
Norfolk et al.	2009	Developing therapeutic rapport: a training validation study	An effective doctor–patient relationship centres on the quality of shared understanding about the nature<br>of the patient's problem and the patient's perspective on their problem. Accurate understanding of presented symptoms is the benchmark of medicine, the primary diagnostic goal – whether that symptomatic pattern is biological, psychological or social at root. Underpinning the now predominant 'patient-centred' approach to consulting, however, is the conviction that an	37 GP trainees participated in a multiphase communication skills training, with 10 other GP trainees participating in a control group.<br>This is a pretest-posttest mixed methods study using a self-created questionnaire, and videotapes of patient consultations.	1) The training group demonstrated significant increases in all three affective dimensions of attitude to rapport, confidence about developing rapport and motivation to develop rapport. In contrast, the control group showed no significant change in any of these dimensions during this time.<br>2) Within the training group, there was significant positive change in rapport-related knowledge over time. Within the control group, there was no	The new training module defined a more clearly identifiable empathic journey, within a model of developing therapeutic rapport. Given that this might initially have appeared complex to new GP registrars, it was particularly encouraging to note the significant increase, over the timespan of the module, in their knowledge, attitudes and confidence associated with rapport.

			equivalent understanding of the patient's experience of their symptoms, their interpretation of what is happening to them, should be seen as a parallel diagnostic goal.		significant rise in scores.<br>3) Within the training group, there was a significant positive change in incidence of positive engagement between consultations assessed before and after training. However, within the control group, there was a significant fall in the rate of positive engagement<br>4) Within the training group, there was a significant positive change in the expert ratings of all aspects of rapport-related behaviour between consultations assessed before and after training. However, within the control group, there was no significant difference between ratings of any of these dimensions at the two time points.	
Ohuabunwa et al.	2017	Towards Patient Safety: Promoting Clinical Empathy Through an Experiential Curriculum in Care Transitions Among the Underserved	Improving patient outcomes is a priority in the practice of Medicine. Experiential learning has been shown to increase empathy and can lead to better patient outcomes.	Medical students took part in a curriculum aimed at promoting patient safety through didactic, experiential, and web-based study. Qualitative analysis of internet blog posts made by the participating students was done to evaluate the outcomes of the curriculum.	Students identified lapses during the transition of patients from inpatient to outpatient. These lapses were either at the system-level, provider-related, or patient-related.	Experiential learning and self-reflection can develop empathetic insight among students. The combination of face-to-face and a web-based approach provides wider exposure to the topic of interest through discussion. Furthermore, the use of a multimodal approach reduced the need for face-to-face

						teachings while still achieving the goals of the curriculum.
Ozcan et al.	2012	The effect of a structured empathy course on the students of a medical and a nursing school	Better health professional-patient communication and relationship can lead to better patient care and outcomes. Empathy is central to this communication and relationship and is beneficial to health professionals too. The reported decline in empathy should be prevented and courses need to help them develop a humanistic view of patients and understand their emotions.	257 Year 1 students participated in an empathy course including 10h of lectures on empathy and communication skills. This is a pretest-posttest quantitative study where the final 143 medicine and 83 nursing students completed the Empathic Communication Skill (ECSS) and Empathic Tendency Scale (ETS).	ECSS and ETS scores increased significantly. Females' ECSS score was significantly higher than that of males initially, but no significant difference was found after the intervention. There is no difference between the ETS scores of females and that of males.	Empathy can be developed regardless of gender through an appropriate training environment. Students' cognitive empathy (assessed by ECCS) and attitudes about empathy (ETS) have improved through application of new teaching and learning in the short term. To achieve an overall dimensional improvement of empathy, the undergraduate curricula need to be reviewed, restructured and amended. Targeted approaches and different modalities can be applied to foster empathy.
Pacala et al.	1995	Aging game improves medical students' attitudes toward caring for elders	It has been suggested that increased empathy is necessary to improve students' attitudes towards elderly patients. Responding to this need, the Aging Game was designed to heighten students' empathy for elderly persons and to stimulate interest in learning to care for them.	39 fourth-year medical students participated in a 3h role-playing elderly simulation game, with another 16 fourth-year students acting as the control group<br>This is a pretest-posttest mixed methods study utilising a self-created instrument containing 57 items designed to assess four domains that are relevant to education in geriatrics: empathy for elderly persons, attitudes toward caring for elderly patients, knowledge of	Participants significantly improved in empathy, attitudes towards caring for elderly patients, and knowledge of geriatrics.	The Aging Game is a potentially effective tool for improving medical students' attitudes toward caring for elderly patients.

				geriatrics, and beliefs about elderly people (7 items were derived from Maxwell and Sullivan's 1980 questionnaire, while 32 items were from Aging Semantic Differential (ASD) of Rosencranz and McNevin (1969)), as well as student evaluations.		
Pacoe et al.	1976	Training medical students in interpersonal relationship skills	While numerous interventions to improve the doctor-patient relationship in medical students have been implemented, most do not include a control group and none of them are based on the Rogerian core facilitative qualities model, which is the most thoroughly researched model for using and teaching interpersonal relationship skills.	13 first year medical students took part in a course for 2 1.5hr sessions per week for 16 weeks involving 16 role playing sessions with pre-post measures of interpersonal skills and personality variables. 7 other students acted as a control group. The Wells Empathic Communication Test, Index of Facilitative Discrimination, Personal Orientation Inventory were used to measure outcomes.	1) The test group achieved a significantly greater level of expressive empathy than the control group<br>2) The test group scored significantly higher than the control group in identifying empathetic responses<br>3) The test group had more gains in the personality areas of self-acceptance and existentiality followed by feeling reactivity and synergy compared to the control.	The results of the study show that interpersonal relationship skills can be taught, and the comparison with the control group suggest that these gains are not simply due to maturation. Furthermore, the effects of the training extend beyond communication skills and affects personal beliefs and attitudes of the participants.
Patel et al.	2019	Curricula for empathy and compassion training in medical education: A systematic review	Empathy and compassion are foundational elements of the practice of medicine and vital cornerstones of high-quality health care. Both are essential in the care of patients, in that empathy (i.e. understanding of patient suffering) is required to spur	A search on a combination of standardised terms and key words (including empathy, compassion, and derivations thereof) was conducted on databases included CENTRAL, MEDLINE,	Majority of studies found the tested training curriculum improved physician empathy or compassion as measured on at least one outcome measure. Training methods involving actual patients (six studies), as well as	The authors identified the following behaviours may improve patient perception of provider empathy and compassion: (1) sitting (versus standing) during the interview; (2) detecting patients' facial expressions and non-verbal cues of

			compassion (i.e. the emotional response involving action aimed at alleviating patient suffering). Previous studies have demonstrated that empathy and compassion decline during both medical school and residency training, with more recent studies now bringing this empathy and compassion decline into question. However, there is currently no standard for empathy/compassion training and thus there is an urgent need to further develop evidence-based training curricula, which can be implemented during medical training, as well as help inform currently practicing physicians.	EMBASE, and CINAHL. All clinical studies of educational curricula that were described as either empathy training or compassion training.	video recording of interviews (four studies), had the highest success rate with 100% of these curricula demonstrating improvement on at least one outcome measure.	emotion; (3) recognizing and responding to opportunities for compassion; (4) non-verbal communication of caring (e.g. facing the patient, eye contact); and (5) verbal statements of acknowledgement, validation, and support. In addition to didactic lectures, incorporating a curriculum in which physicians can practice learned skills might be the best for enhancing physician empathy and compassion. Specifically, like medical training in which clinical skills are practiced in real time with actual patients and oversight by practicing physicians, incorporating similar methods to "practice" compassion in the clinical setting appears to be beneficial.
Pedersen	2010	Empathy development in medical education--a critical review	Petersen 2010 identified that there is a lack of clarity in the literature at describing the possible influences of the dominating elements of medical education and the possible relations between empathy and other aspects of clinical understanding.	This is a systematic review of medical literature that study empathy development in medical education. This review explores some common challenges and assumptions in the literature on empathy development. The philosophical perspective from which the critique is formulated is	194 articles were found initially. After screening by the author, more than 120 were selected for the final review.<br><br>1) How the acquisition of medical knowledge and paradigms may influence physicians' empathy are often neither explored nor discussed in the literature on empathy development in	While a strong focus on biomedical information is essential for better patient outcomes, neglecting the patient's inner state will inhibit the physician's understanding of the patient. Attempts at increasing empathy in medical students need to bridge the gap between biomedicine and the humanities rather than isolate

				predominantly hermeneutics (Gadamer 1989; Heidegger 1997).	medical education.<br>2) Most studies dichotomise the natural science and human sciences, leading to the "soft" approaches being treated as secondary in medical education.	it from other aspects of clinical understanding.
Pelaez et al.	2018	Implementing a medical student interpreter training program as a strategy to developing humanism	Research has shown that when physicians provide humanistic care, patients are more likely to adhere to medical advice and treatment options, resulting in improved health outcomes. Similarly, cultural competency, defined as "a set of congruent behaviours, attitudes, and policies that come together in a system, agency, or among professionals that enables effective work in cross-cultural situations", has been identified as a major determinant of patient satisfaction, adherence, and outcomes. Accordingly, it is imperative that medical education curriculum provide students with activities that will aid in preservation of empathy and humanism,<br>and increase cultural competency, while preparing future physicians for understanding social,	80 first-year to third-year medical students participated in an 8h medical interpretation training program.<br>This is a pretest-posttest quantitative study utilising a post-training assessment survey.	1) As a result of the training, almost all students indicated that they felt more confident with regards to interpreting<br>2) The great majority of students also felt more empathetic towards LEP patients and towards English-only providers, indicating greater empathy for individuals affected by the communication barrier (patients and providers).<br>3) Additionally, students reported feeling more inclined to advocate for patients and to work for system change.<br>4) As a group, the overall students' scores in multiple choice questions about the medical interpretation process and the interpreter's role were significantly improved as a result of the training.	Implementation of medical interpreter training programs can be a successful strategy to develop of humanism in medical students and can aid in the development of culturally versed and empathetic physicians to provide better care for diverse populations.

			economic, and cultural factors that may affect the quality of the care they will provide.			
Poole & Sanson-Fisher	1980	Long-term effects of empathy training on the interview skills of medical students	There is growing concern on deficiencies in physician-patient communication and empathy is important to be sensitive the patient's feelings, communicate that understanding to them and build rapport. However, this skill is not acquired during undergraduate medical education.	25 Year 5 students in the experimental group had participated in an empathy training in Year 2. The program had eight 1.5-2h audiotape-led sessions in groups of 10-12. They were assessed with the Accurate Empathy Scale through a 15-minute patient interview before and after the training. As follow-up, their results were also compared to a control group of 20 Year 5 students who also assessed with the Accurate Empathy Scale, 3 years after the training.	There was a significant increase in empathy level before and after training but a significant decrease from post-training to follow-up, though still significantly higher than that at pre-training.<br>The experimental group had significantly higher empathy levels than that of the controls at follow-up.	Effects of empathy training in preclinical years continue to have some impact three years later. This study further supports that the apprenticeship style of clinical training does not teach nor maintain good physician-patient communication skills. Their clinical experience helps them become more confident history-takers but does little to reinforce previously acquired empathy skills. Hence, communication skills need to be explicit throughout undergraduate medical education and train clinical teachers to be appropriate role models for students.
Potash & Chen	2014	Art-mediated peer-to-peer learning of empathy	Making art is integrated in medical education for experiential learning to initiate transformational change that prompts self-awareness and empathy.	14 medical students participated in a response-art workshop This is a pretest-posttest mixed method study utilising the Relational Aesthetics Questionnaire, the State Empathy Scale and participated in independent small group and facilitated large group discussion pertaining to	Results from the State Empathy Scale revealed that student increased their present state of empathy. Themes identified include the fostering of empathy (for the patient or the medical student artist who created the art), appreciation of holistic care, and the importance of doctor-patient relationship.	Making art may offer medical students an opportunity not only for increased awareness regarding their own and their patients' emotions, but also to educate each other on the important role that empathy plays in the medical profession. One student's learning may become the impetus for another student, thereby forging a connection between peers.

				observations, themes and discoveries.		
Potash et al.	2014	Art-making in a family medicine clerkship: how does it affect medical student empathy?	Many others feel that empathy complements and facilitates successful clinical consultations and quality of care. By creating art and poetry in response to patient situations, practitioners can come to a personal and intimate understanding of various illness experiences, as well as, expanded perceptions. Within medical training and professional development, reflections on the arts encourage multiple perspectives that can help to provide insight and awareness as to how a patient experiences pain and suffering	48 third-year medical students participated in an arts-making workshop with 58 students participating in a problem-solving workshop as a control group.<br>This is a pretest-posttest mixed methods study with a control group utilising the Revised Jefferson Scale of Empathy – Student Version and the impact of the arts-making workshop was explored through themes identified from the poetry, art, and reflective essays produced.	JSE results showed decreases in empathy from the beginning to the end of each rotation.<br>Themes identified included: benefits of an arts-making workshop (reflection, 'humanistic side', clarity, 'enlightening', relaxing, develop creativity and imagination), empathic understanding gained (self-awareness, patient awareness, understanding pain and suffering, role of the doctor).	The authors offered mixed findings on the role of an arts-making workshop for enhancing JSE given the differing quantitative and qualitative results, but observe that it affects emotional awareness, thus suggesting a promising role for such an activity to complement related teaching, rather than serve as a stand-alone component within the clinical clerkship.
Razavi et al.	2003	How to optimize physicians' communication skills in cancer care: results of a randomized study assessing the usefulness of post-training consolidation workshops	Effective communication skills has been shown to produce better patient outcomes. While communication skills training has been shown to be beneficial for physicians, new evidence suggests the adoption of learner-centred, skills-focused, and practice-oriented techniques can complement traditional workshops.	59 physicians took part in a basic training workshop on communication skills. 29 took part in a consolidation workshop after that while 30 did not. Both groups were assessed before the basic training and after the first group completed the consolidation workshop. The assessment consisted of a simulated and actual patient interview. Before the interviews,	1) Physicians who completed the consolidation workshops asked more open and directive questions, used significantly more functions alerting patients to elements of reality they had not perceived before, and made significantly fewer premature reassurances.<br>2) Those who completed the consolidation workshops made more acknowledgments, more empathic statements, more	While a traditional communication skills workshop is effective at improving the communication skills of physicians, having a consolidation workshop as an adjunct can optimize the outcomes further, suggesting that consolidation workshops can help with the transfer of these skills to clinical practice, allowing for patients to perceive these improvements.

				participants completed a sociodemographic questionnaire, the Hospital Anxiety and Depression Scale and the Ways of Coping Checklist. After the interview, the patient and physician competed the Perception of the Interview Questionnaire.	educated guesses, and more negotiations<br>3) Compared to the assessment before the basic training, both groups showed improvements in communicating and displayed more emotion during actual patient interviews.<br>4) Patients reported that physicians who took part in the consolidation workshops reported appeared to have a better understanding of their disease.	
Riess et al.	2011	Improving empathy and relational skills in otolaryngology residents: a pilot study	Empathy and relational skills are critical factors in predicting quality of care, patient safety, patient satisfaction, and decreasing malpractice claims. Physician empathy has been shown to significantly improve health care outcomes, and physicians who express empathy receive higher patient satisfaction ratings. Despite its importance, physician empathy declines throughout medical training. The Accreditation Council for Graduate Medical Education (ACGME) mandates competency in 6 areas, including interpersonal and	11 otolaryngology residents participated in 4.5h neurobiological empathy and relational skills workshop.<br>This is a pretest-posttest quantitative study utilising the Balanced Emotional Empathy Scale, the Jefferson Scale of Physician Empathy, the Neurobiology of Emotions Assessment, the Ekman Facial Decoding Test, and the Consultation and Relational Empathy (CARE) measure.	1) After the intervention, there was statistically significant improvement in residents' knowledge of the neurobiology and physiology of empathy.<br>2) After the intervention, there was statistically significant improvement in residents' self-reported empathy with patients<br>3) Standardized effect sizes were small to medium for physician-rated empathy for patients (CARE)<br>4) Standardized effect sizes were very large for knowledge of the neurobiology and physiology of empathy.<br>5) Residents' ability to decode subtle facial	The authors showed that our empathy training protocol significantly improved surgeons' knowledge of the neurobiology and physiology of empathy, as well as their self-reported capacity to empathize with patients. The residents also showed nonsignificant improvements in their ability to use subtle facial cues to identify emotions and to empathize with others in general. Although not significant, patient ratings of residents' empathy were also in the predicted direction.

			communication skills, which are defined as effective teaming with patients, families, and other health professionals. In addition to mandating interpersonal skills training, the ACGME also mandates evaluation and assessment of these skills.		expressions of emotion (Ekman Test), their self-reported ability to empathize with their patients (Jefferson Scale) and with other people (BEES), and patients' ratings of physician empathy (CARE) all increased in the predicted direction, but none of these effects achieved statistical significance.	
Riess et al.	2012	Empathy training for resident physicians: a randomized controlled trial of a neuroscience-informed curriculum	Delivering compassionate patient-centred care remains a core value of the medical profession and is central to the educational mission of residency and fellowship training programs. However, communication skills training and relational aspects of care have traditionally received less attention and have few metrics for evaluation. Most recent studies show a decline in empathy during medical education that persists beyond training, although there is a recent dissenting view. Empathy has long been mandated as a learning objective for medical school education by the Association of American Medical Colleges, and the Federation of State Medical	54 residents and fellows from surgery, medicine, anaesthesiology, psychiatry, ophthalmology, and orthopaedics, participated in a 3h empathy and relational skills training, while 45 other residents and fellows participated in the control group. This is a pretest-posttest quantitative study utilising the Consultation and Relational Empathy Measure, a self-created Neurobiology and Physiology of Empathy Test, the Ekman Facial Decoding Test, the Jefferson Scale of Physician Empathy, the Balanced Emotional Empathy Scale, and an	1) Physicians in the intervention group showed greater improvement on patient ratings of physician empathy (CARE measure) than did the control group.<br>2) The intervention group showed significantly greater improvements in their knowledge of the neurobiology and physiology of empathy.<br>3) The intervention group showed significantly greater improvements in their ability to decode subtle facial expressions of emotion.<br>4) Differences in self-reported attitudes about the relative importance of empathy in clinical practice (JSPE) did not differ between the groups.	A brief training intervention grounded in the neurobiology of emotions can increase patient-rated empathy among medical and surgical trainees.

			Boards will soon require evidence of competency in physician–patient communication for license renewal.	Attitudes and Skills, and Program Evaluation.		
Rosenthal et al.	2011	Humanism at heart: preserving empathy in third-year medical students	Prior studies have reported that, despite the importance of empathy in doctor/patient interactions, medical education leads to deterioration in empathy among medical students and residents. Clinical and therapeutic advantages of empathetic patient care include improved doctor–patient communication, increased patient satisfaction, greater patient compliance, decreased litigation, increased physician job satisfaction, and decreased physician burnout. Several authors have suggested that educational efforts to enhance humanism and incorporate it into the curriculum may help overcome the perceived decline in empathy during medical school.	209 third-year medical students participated in a longitudinal Humanism & Professionalism curriculum.<br>This is a pretest-posttest quantitative study utilising the Jefferson Scale of Physician Empathy.	1) No significant decreases in empathy scores were detected at the end of the third-year clerkship.<br>2) Gold Humanism Honor Society students had post-test empathy scores significantly higher than the other students in the class.<br>3) Knowledge of selection for the Gold Humanism Honor Society seems to positively influence students' JSPE-MS scores.	Although the authors cannot be certain that the H&P intervention was responsible for the preservation & lack of decline in empathy, student feedback indicated that the sessions helped them "prevent burnout" and recognize positive and negative role models.
Roter et al.	2004	Use of an innovative video feedback technique to enhance	Effective communication skills are important for producing better patient outcomes, and studies have shown that these skills can	28 paediatric residents took part in a 4h intervention spread over 4 weeks focusing on training communication	1) The time that the SP spent on talking increased and the 2nd assessment<br>2) Residents asked more open ended questions at the	1) The results confirms previous findings that females score better than males in the learning of communication skills, and show more

		communication skills training	be nurtured, especially using experiential methods of teaching.	skills using SP interviews and didactic teachings. SP interviews in the first and last weeks of the programme served as pre and post intervention assessments. The RIAS software was used to code video recordings of the SP interviews. Feedback from coding of the first interview was done in week 3.	end of the intervention<br>3) There was a significant in the expression of empathy<br>4) Residents displayed greater willingness to build a relationship with patients	improvements than males.<br>2) The areas of improvement that the female and male participants displayed were different<br>3) Interviews coded with RIAS are acceptable and efficient in providing feedback for improvement<br>4)
Ruiz-Moral et al.	2017	Teaching medical students to express empathy by exploring patient emotions and experiences in standardized medical encounters	A physician's communication skills and empathy can lead to improvements in clinical outcomes. Studies have shown that empathy can be developed.	115 medical students took part in the intervention focusing on using communication skills to obtain information from patients and making shared decisions with patients. The outcomes were measured using an external observer rating the students interviews with SPs as well as SP feedback.	Progressive improvements in communication skills was observed with each SP interview. This was seen by the external observer rating and SP rating.	Communication skills training is vital in curbing the decline of empathy in students and is useful in allowing students to develop a better understanding of patients. Improving communication skills and encouraging students to explore the concerns and feelings of patients is a preliminary step for greater empathic engagement. The results suggest that the "empathic process" is sequential and needs to be taught in a way that is feasible and understandable to students. Students reported discomfort in exploring patient's emotions and experiences, thus it is importance to normalize their reluctance to explore these concerns and reassure them that it is

						necessary in the therapeutic relationship.
San-Martín et al.	2017	Role of a Semiotics-Based Curriculum in Empathy Enhancement: A Longitudinal Study in Three Dominican Medical Schools	Empathy is important to patient care and interventions have shown to be effective in nurturing it. Medical semiotics aim to help global understanding of the treatment process where symptoms and signs need interpretation, and hence promote humanistic medicine.	165 students from 3 different medical schools were compared.<br>School A had a theoretical and practical curriculum, School B had a theoretical semiotic-based curriculum while School C had 20 credits of medical semiotics during the clinical years. They were assessed with the JSE-S.	School A and B had an improvement in empathy but School C (control) had a clear decline.	This study offers empirical evidence which supports that empathy can be improved with relevant education.
Sands et al.	2008	Pediatric narrative oncology: interprofessional training to promote empathy, build teams, and prevent burnout	Empathy, teamwork and resilience can be difficult to teach but narrative competence has been suggested to help. Narrative medicine aims to increase empathy, build teams, strengthen resilience and reduce burnout	19 paediatric oncology HCP (6 doctors) participated in an interdisciplinary narrative medicine training.<br>All participants completed a pre- and post- IRI and SSPON test. A focus group was also conducted with 14 participants.	1) There was a significant increase in ability for Perspective Taking over time<br>2) There was an increasing trend of Empathic Concern, though not statistically significant.<br>3) There were no significant changes in Fantasy or Personal Distress.<br>4) There was a significant increase in the levels of stress reflected by the Death Without Grace score and Co-Worker Incompetence score but not for the remaining domains.<br>Themes from focus groups :<br>1) The seminar revealed new aspects of the self of the individual writer.<br>2) Participants observed that	A brief staff multi-disciplinary intervention in a busy inpatient unit is feasible. This training can increase empathy, helped improved relationships and was reported to be useful and pleasurable across disciplines, despite not reducing stress levels.

					the responsiveness of the writing and sharing process transferred to the interprofessional interactions of team members outside the focus group setting and in the natural work environment.<br>3) Participants observed that the structure and timing of the narrative seminar need to be carefully considered so as not to conflict with clinical duties.	
Sanson-Fisher & Poole	1978	Training medical students to empathize: an experimental study	Studies have shown that the doctor's interpersonal skills and empathy are more important to patients than technical competence.	135 preclinical students (112 test, 23 control) took part in the intervention. A pre-test assessment of their ability to empathize was conducted using a 15min interview with a patient using the Accurate empathy Scale. The intervention group took part in the tune-in empathy training workshop, which consisted of 8 audiotape-led sessions over 8 weeks. All students were then assessed again 8 weeks later, with an additional self- report questionnaire to assess their attitudes about empathy and training programme.	1) The test group had significantly higher empathy scores after the intervention as compared to the control group.<br>2) Students reported a strong positive response to the training programme.<br>3) Patients reported that after the training, the students were understanding and caring, and the students put in more effort to understand the patients better.	The present study has demonstrated that the skill of empathizing can be trained. Empathy training should be essential and should be a prerequisite of medical training.

Schoonover et al.	2019	Impact of Poetry on Empathy and Professional Burnout of Health-Care Workers: A Systematic Review	Physician burnout is a public health crisis, but humanities in medicine may promote empathy and prevent burnout. Narrative medicine can help to experience the perspective of another person, and poetry has been shown to help physicians manage grief, develop compassion and promote empathy. This systematic review reviews poetry and its impact on empathy.	A search on "narrative medicine", "healthcare worker" and "empathy", "burnout" or "occupational stress" was conducted since inception to September 25, 2018. The databases included Ovid MEDLINE(R) and Epub Ahead of Print, In-Process & Other Non-Indexed Citations, and Daily, Ovid EMBASE, Ovid PsycINFO, Ovid Cochrane Central Register of Controlled Trials, Ovid Cochrane Database of Systematic Reviews, and Scopus.	Overall, there is evidence that poetry, part of a narrative medicine intervention, increases empathy, but limited evidence that it reduces professional burnout.<br>Only one research letter utilized poetry exclusively to improve empathy, while none of the included studies used poetry exclusively.	Longitudinal studies with consistent and standardized assessments can be conducted to further investigate the impact of poetry on empathy and burnout.
Schweller et al.	2017	Nurturing virtues of the medical profession: does it enhance medical students' empathy?	Good role modelling is important and reflection with good role models can help medical students cope with the new stressors in medicine. Empathy can develop professional identity.	166 Year 1 medical students participated in a Health and Medicine course on developing their professional identity. There were interviews with physicians, patients, supervised visits to hospitals, videotaped simulated consultations and caricatures of "really bad" consultations.<br>This was a pretest-posttest study using the JSPE-S.	1) There was a significant increase in mean JSPE scores. The effect size was relevant though small.<br>2) The increase in empathy levels was significantly greater for students who had a lower level of empathy at baseline.	Despite empathy not being the core in the intervention, it was still successful in developing empathy.<br>Students with lower initial empathy might have been the most susceptible to the hidden curriculum and negative role models, thus interventions would give them the greatest gains.<br>Starting in Year 1 would be useful to develop a longitudinal follow-up throughout and reinforce ideas of professionalism and personal growth, possibly shielding any future decline.

Schweller et al.	2014	The impact of simulated medical consultations on the empathy levels of students at one medical school	Empathy is important to the doctor-patient relationship and has been shown to be beneficial to patients and physicians. There have been several strategies proposed to improve or preserve empathy in medical students, but there aren't many objective studies in this area.<br>This study describes a simulation with SP and assessed its impact on the empathy of Year 4 and Year 6 medical students.	123 Year 6 and 124 Year 4 students participated in 4 simulation activities with SP and debriefings every week.<br>This is a pretest-posttest quantitative study using the JSPE and IRI. The post-test was within 30 days or after 3 months for the Year 6 and Year 4 students respectively.	Intra-group analysis showed a significant increase in JSPE score and in mean IRI score for the Year 4 and Year 6 students.	Adequate time for reflection on their own experiences and others' experiences is important in developing professional competence and empathy.<br>Having facilitators who practice medicine daily helped to contextualize the topics discussed, hence students won't view the topics as idealized or intangible.
Shapiro & Hunt	2003	All the world's a stage: the use of theatrical performance in medical education	Medical educators have long been interested in the potential of the humanities to enliven and inform various aspects of medical education. The humanities are typically viewed as a means of humanising medical education. The theatre has provided intriguing therapeutic and educational possibilities. Theatrical performances have been introduced as an important aspect of innovative patient care environments, especially in Europe.	70 medical students (about a third), faculty members (about a quarter) and health professionals serving the AIDS community watched a 1h theatrical performance about AIDS.<br>80 medical students (about 20), faculty members, health professionals associated with the Cancer Centre, cancer patients and their friends and families (about a third) watched a 1h theatrical performance about ovarian cancer.<br>This is a qualitative study utilising on-site discussion and informal follow-up feedback	1) The most frequent issue represented in statements made by students either in group discussions or follow-up feedback concerned the importance of developing empathy and compassion for the patient's experience.<br>2) Students seemed to pay great attention to how doctors were portrayed in both performances and mentioned as anti-role models those who were callous or indifferent.<br>3) Students also mentioned the importance of seeing the patient in the context of his or her life, rather than simply reducing them to a diagnosis or treatment challenge.	Theatrical performances as an adjunct method of enhancing empathy towards patients in a self-selected subset of medical students proved highly successful. Those who participated were moved were moved and felt they had gained important insights into the nature of the patient experience.

					4) Event evaluation forms showed extremely favourable reactions.	
Shapiro et al.	1998	Effects of mindfulness-based stress reduction on medical and premedical students	Coping with stress appears to be one of the greatest challenges currently facing the medical profession. The extreme stress levels inherent in the medical profession (and in preparing for it), put premedical and medical students at risk for both physical and psychological problems. Stress may affect not only medical students' personal well-being but may also have negative consequences on their professional effectiveness by diminishing the humanistic qualities fundamental to optimal patient care. However, rather than helping students cultivate empathy, medical school may play a role in decreasing it.	37 medical and premedical students participated in a 17.5h mindfulness-based stress reduction & relaxation elective, with 37 other students acting as a control group.<br>This is a pretest-posttest quantitative study utilising an adapted version of the Empathy Construct Rating Scale, the Hopkins Symptom Checklist 90 (Revised), Subscale 4 of the SCL-90, State-Trait Anxiety Inventory (Form Y)-STAI Form 1, and the Index of Core Spiritual Experiences (INSPIRIT)	The data indicate that participation in a mindfulness-based stress reduction intervention can effectively<br>(1) reduce self-reports of overall psychological distress including depression,<br>(2) reduce self-reported state and trait anxiety,<br>(3) increase scores on overall empathy levels, and<br>(4) increase scores on a measure of spiritual experiences assessed at termination of the intervention.<br>These results (5) replicated in the wait-list control group,<br>(6) held across experimenters, and<br>(7) were observed during the exam period.<br>Further, analysis demonstrated that one's compliance with treatment played an important role in outcome.<br>Scores on the empathy measure increased significantly, suggesting that the intervention may have helped students	This study documents the potential effectiveness of mindfulness training to enhance premedical and medical students' psychological/spiritual well-being as well as help cultivate skills to use in their future roles as physicians.

					cultivate listening skills and develop new, more compassionate perspectives and paradigms to approach their own lives as well as their future patients' lives.	
Shapiro et al.	2006	Point-of-view writing: A method for increasing medical students' empathy, identification and expression of emotion, and insight	Writing or other narratives have the potential to help medical students reflect on their own clinical experiences and enhance empathy, communication skills, professionalism and well-being. However, there is little empirical research. It is unknown whether a writing technique would be more effective than other methods.	92 Year 2 students were enrolled in a doctoring course teaching interviewing skills, PE and clinical reasoning. 47 students were randomly assigned to write about the point of view of the SP or patient after each session while 45 students were assigned to write about their clinical reasoning.<br>Students were evaluated with 3 OSCE cases. SP assessed their performance on 5 communication skills; a global professionalism item and a global satisfaction score on a 5-point Likert scale. The students' essays were analysed with self-developed thematic codes, rated on overall empathy and insight and coded using the Linguistic Inquiry and Word Count (LIWC).	1) Thematic Codes: The POV group's essays were higher significantly more likely to adopt the first person point of view, show empathy for the doctor, and received a higher overall empathy and insight rating.<br>2) LIWC: POV students significantly used more "I" pronouns, more words of emotion, more religious and spiritual references. CR group showed a tendency to use more negative emotion words, words expressing anger.<br>3) SP: There were no significant differences between the 2 groups.	The discrepancy between the SP rating and essay evaluation could suggest that students need more clinically relevant training to effectively express the positive qualities in their writing, and that empathy expression might be case-specific. SPs might also focus more on technical competence than the importance of empathy.
Shapiro et al.	2004	Teaching empathy to first year medical	Empathy is necessary to medical education. If it is as	22 students participated in eight small group	1) There were no significant changes in average ECRS	The standard cognitive-behavioural method of

		students: evaluation of an elective literature and medicine course	much art as science, humanities might help to develop it. While it has been received positively by learners, little is known about its impact on improving empathy and professionalism.	reading and discussion sessions on medical humanities. Emphasis was on understanding and identifying with different points of view.<br>This was a mixed-methods prettest-posttest study using a focus group interview, ECRS and BEES.	scores.<br>2) There was statistically significant increase in average BEES scores.<br>3) Female students, Asian students and students planning to enter primary care had significantly higher post-intervention BEES score.<br>Qualitative:<br>- more complex understanding of the patient's perspective<br>- understand the benefits of literature on helping them become better physicians<br>- useful in coping with training-related stress	teaching did not seem to improve self-reported empathy, reflecting existing concerns on the most appropriate way to teach empathy.<br>Nonetheless, this intervention reflected that literature can be effective in nurturing some aspects of empathy and improve attitudes toward humanities.
Shapiro et al.	2019	The human kindness curriculum: An innovative preclinical initiative to highlight kindness and empathy in medicine	Physicians 'empathy has shown to be correlated to their ability to cope with stress at work, but exposure to the medical humanities which could promote compassion and empathy has been absent from many medical school curricula or is only a voluntary elective. Studies on empathy-enhancing interventions have failed to see the long-term impact of such interventions on student.<br>This paper describes a compulsory human kindness (HK) curriculum aimed at strengthening kindness,	138 Year 1 (Group 1) and 2 (Group 2) medical students participated in the pilot study. The course was improved and modified according to the outcomes and students' feedback. In the second year, 101 original Year 1 students who were now in Year 2 (Group 3) and a new intake of 104 Year 1 students (Group 4) participated in the revised HK curriculum. The modalities included humanities, arts, theatre exercises, narrative medicine, neuroscience of	1) There was no significant change in the JSE scores of Group 1 and Group 2 and JSE scores even declined.<br>2) There were significant improvements in Group 3 and 4.<br>3) Females had a higher self-reported empathy score.<br>4) Narrative comments increased, and positive comments decreased from Group 1 to Group 3.<br>Students enjoyed the clinical relevance of the intervention but doubted if they could be "taught" kindness.	There is a gender difference in self-reported scores. It is difficult to compete with the hard sciences for preclinical students' attention; hence it is important to be innovative and demonstrate clinical relevance, which would allow students to perceive it as useful.<br>The curricular change to focus on more clinical exposure and applications improved the effectiveness of the curriculum, particularly for those who had a low baseline JSE score and maintained empathy levels among those with initially

			compassion and empathy in pre-clinical medical students.	empathy, loving-kindness meditation, virtual and SP and behaviourally based videotapes.<br>This was a pretest-posttest quantitative study using the JSE. Medical students also rated each session on a 5-point Likert scale and gave narrative comments.		high scores. It also prevented the frequently reported decline in empathy.
Sin et al.	2019	Evaluation of Constructing Care Collaboration - nurturing empathy and peer-to-peer learning in medical students who participate in voluntary structured service-learning programmes for migrant workers	Community service has been shown to provide numerous benefits for the volunteer. Studies have shown that in the medical setting, volunteering can lead to better patient outcomes.	Medical students took part in a community service project aimed at providing basic medical care for migrant workers.<br>A mixed methods approach was adopted to collect data from the participants regarding their views of the project and its impact on their communication skills and empathy.	Participants reported an increase in all domains of the Fund for the Improvement of Postsecondary Education questionnaire as well as improvement in their communication skills.<br>Students reported an increased understanding of public health measures and were more likely to think about the future consequences of patients.<br>Students reported a better understanding of migrant workers and felt an increased level of empathy towards them.<br>Students reported increased social awareness towards migrant workers as well as the issues they face when navigating the healthcare system.<br>Students enjoyed the	The use of volunteer manpower significantly reduces financial costs of interventions while still providing benefits to all volunteers. Experiential learning with a focus on reflection and group discussion can lead to better outcomes for participants.<br>Peer-to-peer teaching provides a safe platform for learning and for both the teacher and the student and may play a part in improving empathy.

					opportunity for peer-teaching in the programme.	
Singh et al.	2017	Low-fidelity simulation to enhance understanding of infection control among undergraduate medical students	Based on limited data, developing countries report higher HAI rates compared with those in Europe or the USA and this gap widens further in intensive care units. Lack of adequate knowledge coupled with poor adherence to infection control protocols is a universal phenomenon among medical students.	A randomised control trial was implemented for 102 second year medical students with the cohort split into the control, video show, and case discussion group. The Toronto Empathy questionnaire was used before and after a course on infection control.	Pre-test TEQ scores were similar in all 3 groups. After the course, the case discussion and video show groups had significantly better TEQ scores than the control group.	The authors suggest that their qualitative results provide the basis for change in the culture towards better adherence to infection control measures.
Singh et al.		Using Role-plays as an Empathy Education Tool for Ophthalmology Postgraduate	Soft skills are an important part of medical education that has largely been neglected. Empathy is among these skills and has benefits for both the patient and physician.	39 ophthalmology residents took part in a empathy sensitizing module that uses role play to increase empathy among participants. The JSPE was administered before and 6 weeks after the programme. External evaluators also assessed the behaviour of these participants in the work environment.	1) An improvement in self-rated empathy scores was observed.<br>2) Externally rated empathy and soft skills also improved.<br>3) Focus group discussion revealed that the skills learnt during the programme was slowly lost due to a lack of reward/credit for effective communication. The infrequency of training, patient overload, and lack of continuous assessment also contributed. Negative role modelling acted as a barrier for continued practice of good communication.	Role playing is an effective tool to improve empathy, but it should be introduced early in residency and must be contextualised in real life scenarios.
Smith et al.	2017	The complexity of empathy during	Previous studies	129 medical students took part in the study where	JSE scores showed a significant decline over time	The discrepancy between JSE and QCAE scores show the

		medical school training: evidence for positive changes	suggest that empathy declines during medical training. However, this past research relied on a single narrowly operationalized self-report measure of empathy.	they completed online surveys and computerized tasks to assess empathy. These assessments were conducted at the beginning and the end of each academic year for the first 3 years of medical school.	while QCAE scores increased over time.	importance of assessing both cognitive and affective empathy. Medical school does not lead to an overall decline in empathy, but the changes are more complex with some aspects of empathy increasing over time.
Son et al.	2018	Communication skills training and the conceptual structure of empathy among medical students	Physicians' empathy for patients is crucially important for gathering appropriate information from patients and for understanding patients' illnesses and suffering. Empathy enables a physician to provide quality patient care without becoming too emotionally involved. Moreover, empathy appears to be associated with personal and professional growth, well-being and career satisfaction. Finally, empathy is positively related to patient satisfaction, increased compliance to treatment and fewer complaints of malpractice by patients. Some studies suggest that a significant decline in empathy occurs during the clinical training years in medical school as documented by self-assessed	295 fourth-year medical students participated in communication skills training.<br>This is a pretest-posttest mixed methods study utilising the Jefferson Scale of Physician Empathy, a self-created three-item scale to measure students' willingness to show empathic behaviour and fit in the context of the training, as well as an open ended question on how the training session and OSCE influenced their communication skills in terms of empathy.	1) Although for both perspective taking and compassionate care the correlation with willingness to show empathic behaviour was a bit higher for post-training than for retrospective pre-training, the correlation between perspective taking and willingness to show empathic behaviour was on both occasions substantially higher than the correlation between compassionate care and willingness to show empathic behaviour.<br>2) 16 themes were identified, with 12 themes on positive aspects of learning and 4 themes on difficulties of learning. Taken together, these findings from the open-ended responses appear to indicate that students experience difficulties in empathizing with patients	The authors' findings indicate that students' willingness to show empathic behaviour is much more correlated with perspective taking than with compassionate care.

			empathy scales along with an increased cynicism and emotional exhaustion in later years		emotionally yet do manage to acquire the cognitive skills that are needed to communicate with patients.	
Sripada et al.	2011	A randomized controlled trial of a feedback method for improving empathic accuracy in psychotherapy	The possibility of error and bias in understanding the perspective of the other, both on the part of the therapist and the patient, is now widely recognized. Because of the transactional nature of empathy, inaccuracy may occur because of patient or therapist bias.	16 patients and 12 psychiatry residents participated in a feedback method for improving empathic accuracy in psychotherapy, with 10 patients in the experimental arm and 6 patients in the control arm.<br>This is a quantitative study with a control group utilising the Patient Global Assessment of Function, the Therapist's Estimate of the Patient's Global Assessment of Function, and the Barrett-Lennard Relationship Inventory.	1) The feedback intervention can improve empathic accuracy, as measured by the extent to which the therapist could predict the patient's self-rated GAF.<br>2) BLRI showed that the therapists with feedback intervention have higher empathy scores.<br>3) Patients with feedback intervention showed decreased distortions (more accurate perception of the therapist's empathic accuracy)<br>4) Therapists in the intervention condition showed evidence of lower levels of over or under confidence in their ability to accurately estimate their patient's self-ratings	The authors describe a feedback method, which with minimal effort increased the accuracy of the therapist's view of patient's experience.
Srivastava et al.	2017	Teaching clinical empathy to undergraduate medical students of Dehradun: A quasi-experimental study	The decline of empathy can be prevented by targeted interventions. Empathy is a skill that still requires more attention in India, hence this study aims to evaluate if empathy can be changed through education in an Indian context.	Years 1, 2 and 3 medical students participated in an interactive audio-visual teaching session on clinical.<br>This was a pretest-posttest quantitative study using the JSPE-S. Data from 328 students was analysed.	There was a statistically significant increase in mean empathy scores and the effect size was large. This improvement was irrespective of age, gender, year of education and specialty interest.	This study adds to existing quantitative and qualitative studies on empathy interventions.<br>This study shows that specially designed empathy intervention can be effective in improving empathy regardless of age, gender,

						year of study and specialty interest in the Indian context.
Stebbins	2005	Enhancing empathy in medical students using Flex Care(TM) communication training	Empathy can be improved through education intervention and plays an important role to physicians and patients. However, medical students have been reported to lose their ability to empathize through pre-clinical and clinical curriculum. Flex Care(TM) is an interpersonal communication training programme developed from the Myers-Breiggs Type Indicator. The author aims to test the ability of this programme to enhance empathy amongst second-year osteopathic medical students	65 randomly assigned Year 2 medical students participated in the FLEX CareTM training programme, which is a specific application of the MBTI and designed to enhance interpersonal communication in a healthcare setting. The intervention had four 2-hour weekly sessions. After the intervention, 106 students (53 in the experimental group and 53 in the control group) were assessed with the JSPE and by standardized patients using the Standardized Patient Feedback Form - Part II.	1) There wasn't a statistically significant difference between the experimental and control group on their sum JSPE scores. The control group's mean score was slightly higher than that of the experimental group.<br>2) There was no significant difference between the experimental and control groups performance on the Standardized Patient Feedback Form - Part II.<br>3) There was no statistically significant correlation between the JSPE and SPF.	The intervention has helped provide students with a communication model in the healthcare setting to improve their behavioural empathy to not just understand but also meet the needs of the patient. There is no single quick fix to reverse the decline of empathy in medical students. A formal curriculum must be developed to embrace behavioural and social aspects of medicine and counteract the powerful effect of medical education and socialization. Students should be taught self-discovery to help them balance the intense focus of only the science of disease.
Stebletsova et al.	2017	Empathy development through ESP: A pilot study	Medical practitioners appear to have a special status in any society. In exercising their professional skills and abilities, they are expected to reveal high standards of morality and ethics. Empathy is an important ethical component in medicine that makes a considerable contribution to an overall clinical outcome. Medical educators consider empathy to be a crucial aspect of healthcare which is	128 first-year general medicine, paediatric, pharmacy, dentistry and nursing students participated in an English for Specific Purposes course that incorporated empathy-developing content.<br>This was a pretest-posttest quantitative study utilising the Balanced Emotional Empathy Scale.	The results have demonstrated considerable improvement of empathic abilities in all specialty and gender groups	Even a slight shift of the teaching focus from professional topics only (for instance, diagnosing, treatment, prevention) to both professional and ethical issues (for instance, doctor-patient communication) could contribute significantly to empathy development through the use of the English language classroom.

			due to be developed in the course of medical education.			
Stepien & Baernstein	2006	Educating for empathy. A review	Medical educators have an interest in promoting empathy in their trainees. As a component of the physician-patient relationship, empathy affects both diagnosis and patient care. Patients who feel listened to are more likely to fully explain their symptoms and to provide pertinent details. Emotional as well as intellectual engagement may help physicians attend to aspects of patients' health that might otherwise go unnoticed. Physician empathy may also significantly influence patient satisfaction, adherence to medical recommendations, and medical-legal risk.	A search for studies that address the effectiveness of strategies for teaching empathy to medical students (Medical Subject Heading terms education, medical, undergraduate or student, medical crossed with empathy) was conducted using PubMed. 13 peer-reviewed English language, qualitative and quantitative studies reporting primary data on interventions that aim to foster empathy in undergraduate medical student were identified.	6 studies focused on the behavioural dimension of empathy, approaching empathy as a communication technique.<br>4 studies utilised medical literature courses, participated in reflective writing exercises, or attended theatrical performances.<br>2 studies describe an approach to promoting empathy in which students experience medical care from a patient's perspective.<br>1 large cross-sectional survey investigated whether prior education in empathy, spirituality, or wellness during medical school was correlated with higher empathy scores.	Brief, targeted interventions can have major and lasting impact on student's ability to display empathy in patient interactions. Educators should be encouraged to incorporate empathy into medical student courses devoted to communication skills and professionalism.
Sweeney & Baker	2018	Promoting empathy using video-based teaching	Empathy is fundamental to patient-centred care. Effectiveness of interventions to improve empathy has been mixed and there is a lack of consensus on the most effective approach.	48 Year 1 medical students watched videos of the interview of 4 patients about their hospital experience, followed by a facilitated discussion.<br>The sessions were evaluated using the Kirkpatrick framework. Students also reported their satisfaction on a 5-	1) Students enjoyed the originality and interactivity of the sessions.<br>2) Of the 39 paired PPOS responses, 54% had an increase in patient-centred attitudes. There was an increase in mean score.<br>3) Thematic analysis of students' feedback reported positive communication	Group/discussion-based learning allows challenging of each other's perspectives and reflection can help develop non-cognitive skills.<br>Using multimedia content in education help make the intervention more reproducible and promote a better learning experience for the students as less face-to-

				point Likert scale and free-text feedback. They also completed a pretest-post-test PPOS survey.	changes and approach to patients.	face contact could allow for more honesty.
Thepwiwatjit et al.	2019	Patient interviews improve empathy levels of preclinical medical students	Empathy is important to patient care, but empathy level tends to decline in medical school, hence empathy maintenance and improvement is emphasized.	325 Year 2 medical students participated in a Humanistic Medicine course involving first-hand patient interviewing and subsequent discussion and reflection writing.<br>This was a prettest-posttest study using the JSPE-S. 296 questionnaires were complete.	There was a significant increase in JSPE scores. Students with a lower baseline score had a significantly higher improvement.<br>There were correlations between GP and improvement.	This intervention is unique in involving authentic patients and supports existing literature that experiential learning is effective in improving empathy.<br>It was particularly effective in students with lower baseline empathy levels.
Tsao & Yu	2016	There's no billing code for empathy - Animated comics remind medical students of empathy: a qualitative study	Empathy is an essential component of patient care with benefits for both the patient and physician. Studies have shown a decline in empathy throughout training, yet studies have shown that empathy can be taught.	The use of humanities in medical education is well documented. While teaching with comics is a novel idea, it is based on the Kolb's experiential learning cycle.<br><br>This is a mixed methods study involving 25 Year 1& 2 medical students in a Canadian university. Students individually viewed 2 online comic strips on diabetes management and were invited for focus group discussions as well as empathy measurement via JSPE 1 week after the comic strip was viewed.	1) Qualitative findings:<br>a) Most participants were aware and concerned about empathy decline<br>b) Students provided some barriers to maintaining empathy<br>c) Students suggested some strategies to stop empathy decline<br>d) Most students reported that the comics increased their knowledge of the patient perspective, emphasized the importance of empathy, and served as a reminder of empathy<br>e) The comics also enhanced their communication skills	The authors conclude that comics can be an effective tool to help student gain knowledge, change attitudes towards patient encounters, improve observational skills and reflect on empathy and patient encounters.

					2) Quantitative findings<br>a) JSPE scores trended up over time but were not statistically significant for time, gender, or year of study effect	
Tulsky et al.	2011	Enhancing communication between oncologists and patients with a computer-based training program: a randomized trial	The authors designed an interactive, computerized, tailored intervention targeted to improve oncologists' communication skills, which would address the needs of it to be easily accessible, not disruptive to clinical practice, inexpensive and brief.<br>The aim of this study is to test the effectiveness of this intervention in improving oncologist behaviour, and to evaluate the effect of the intervention on patients' perceptions of their oncologists.	This is a single-blind, randomized, controlled, parallel-group study. 48 oncologists attended a 1-hour lecture on communication skills. Of which, 24 oncologists in the intervention group were given a CD-ROM communication skills training program based on their own audio-recorded clinic visits to review over 1 month.<br>This intervention was based on social cognitive theory and a barriers model proposed by Cabana et al.<br>Outcome measures were from the audio recordings of the oncologist's interaction with patients. The number of empathic statements and continuer responses to empathic opportunities were counted. Patients were also surveyed on their level of trust towards the oncologist, perceived level of physician	1) The mean number of empathic statements per conversation increased in the intervention group compared to the control group.<br>2) Patients of the intervention group reported higher trust, greater perceived empathy, greater sense that they were understood as "a whole person"	The authors recognize the importance of individualized, reflective feedback in teaching communication skills. This study provides support that their short intervention could be another good alternative to the gold standard of providing such feedback over a long course with small-group teaching.<br>This study also supports that a communication skills intervention on physicians could improve patient-reported outcomes.<br>This intervention could be expanded to more oncologists for a relatively lower cost, and applied to other specialities once it is refined. It could be easily implemented (recordings could be done on smartphones) and the feedback process could be automated as well.

				empathy, perceived knowledge of patient and therapeutic alliance.		
van Dijk et al.	2017	Effects of Mindfulness-Based Stress Reduction on the Mental Health of Clinical Clerkship Students: A Cluster-Randomized Controlled Trial	Medical students are at a greater risk of psychological distress as they progress through medical school. MBSR has been shown to be useful in reducing distress and burnout in students.	A cluster randomised control trial was conducted involving 167 out of 232 first year medical students (83 intervention, 84 control). Students completed a baseline assessment using the BSI, MHC-SF, LiSat-9, JSPE, FFMQ, and IBI. Assessments were repeated 3, 7, 12, 15, and 20 months after baseline. The intervention group took part in 8 weekly 2h sessions of MBSR while the control group had clerkships as usual.	1) Students in intervention group reported lower levels of psychological distress, increased life satisfaction, more mindfulness skills, and less dysfunctional cognitions than those in the control group.<br>2) No difference in empathy was noted between the 2 groups	1) MBSR integrated into clinical clerkships are feasible for medical students and are an effective way to produce consistent long-term improvements in mental health<br>2) MBSR addresses core competencies in CanMEDS and should be a valuable component of medical curriculum to develop balanced professionals.
van Vliet et al.	2017	Long-term benefits by a mind-body medicine skills course on perceived stress and empathy among medical and nursing students	Empathy is important to the patient-provider relationship, patient care, and a CanMED core competency but it has been shown to significantly decline in medical students overtime. It might be due to increased stress and burnout. A mind-body medicine course has been developed to overcome the challenges in clinical practice and enhance psychological and physiological functioning.	74 medical students and 47 nursing students (intervention groups) participated in an 11-week Mind-Body Medicine course including meditation, relaxation, guided imagery etc.<br>This is a pretest-posttest quantitative study. The intervention and control group (61 medical students, 64 nursing students) took a questionnaire (Perceived Stress Scale, IRI,	Medical students:<br>The intervention group had significantly higher fantasy and empathic concern than the control group at T1 and T2.<br>Personal distress in the intervention group was significantly lower than the control at T3.<br>Within-group:<br>Participating medical students had significantly higher scores in fantasy and nearly in empathic concern after the intervention. They had a significantly higher	This MBM course has resulted in long-term beneficial effects in medical students (reduced stress and favourable changes in empathic concern.)<br>Existing empathy interventions in medical schools have focused on influencing the cognitive component, but the increased empathic concern from this MBM course has shown that it can also influence the emotional component of empathy as it focuses on the enhancement of the self.

				Groningen Reflection Ability Scale) at baseline (T0), right after the course (T1), at 6 months (T2) and 12-months follow-up (T3).	GRAS score from baseline to T2.	
Van Winkle et al.	2012	Impact of a workshop about aging on the empathy scores of pharmacy and medical students	Empathy is important in HCP-patient relationship which has been shown to be related to optimal patient outcomes. However, empathy has been reported to decline through medical school, and postgraduate medical education. There have been reports of different approaches to improve empathy among health profession students, but their short-term and long-term effects have not been documented with a psychometrically sound instrument specific to empathy.<br>This study aims to determine the immediate and sustained impact on empathy scores of a workshop of pharmacy and medical students.	During a 40-minute workshop, 187 Year 1 pharmacy and 183 Year 1 medical students watched a 10-minute performance on the challenges of aging followed by a 30-minute group discussion on the performance.<br>The JSE was administered to all participants pre-test, right after the workshop (post-test 1), and 7 and 26 days after the workshop for pharmacy and medical students respectively (post-test 2)	The increase in empathy scores of pharmacy and medical students increased after the intervention, but it was not sustained.<br>No significant correlation was found between empathy scores and gender or specialty interest.	This intervention was uniquely short, and different from the typical intervention on aging which involves role-play.<br>The findings are encouraging, but the duration can be expanded in future studies to account for the small effect estimates in this current study.
Varkey et al.	2006	The Aging Game: improving medical students' attitudes toward caring for the elderly	There will be greater demands on geriatric medical care, but there is still a large gap in geriatric medicine education. The Aging game was introduced to allow students to	84 Year 1 students participated in an aging simulation experience for 3 hours in groups of 10-12.<br>This is pretest-posttest quantitative study using	There was a statistically significant change in 6 of the 8 attitudes towards caring for the elderly, and a statistically significant increase in empathy towards elderly patients.	It is important for medical students to be exposed to elderly patients earlier on as exposure is limited and inexperience might overwhelm them. The authors hope that this experiential

			personally experience aging and develop empathy for the elderly patients.	the Aging Semantic Differential (ASD) to assess general attitudes toward the elderly and an 11-question modified Maxwell and Sullivan questionnaire to assess participants' empathy and attitudes toward caring for the elderly. Students also evaluated their receptiveness towards the intervention on a 4-point Likert scale.	There was a statistically significant change in 23 out of 32 questions in the ASD. Open-ended comments from students indicated that they valued the experience	learning could create a more lasting impact on the students. A simulation experience with discussions can be effective in improving empathy and attitudes toward caring for the elderly.
Wang et al.	2018	Graphic Stories as Cultivators of Empathy in Medical Clerkship Education	Empathy is an essential component of patient care with benefits for both the patient and physician. Studies have shown a decline in empathy throughout training, yet studies have shown that empathy can be taught.	Medical students took a baseline JSPE assessment before reading case vignettes and comics of those cases. Structured interviews were conducted a week later to obtain qualitative feedback and assessment of the student's empathy.	Students valued empathy highly and recognised it as an essential skill in the doctor patient relationship. Students had differing ideas of what empathy means and whether it can be taught. Students reported different trajectories of empathy that they had experienced and stated some barriers and facilitators to empathy that led to these trajectories. Students showed an understanding of burnout and provided some strategies to curb burnout and promote empathy. In general, students received the comics positively	The cognitive component of empathy was undermined by the "hidden curriculum" in medical education, the behavioural component is heavily influenced by role models during clerkship, and the affective component is influenced by environmental, psychological, and personal barriers to empathy. As such, targeted interventions for each component of empathy are more effective. Reflective practice is useful in empathy development and a repetitive, longitudinal approach to empathy education can promote long term retention of empathy. The stigma surrounding mental health acts as a barrier for student wanting to seek help and efforts should be made to

						allow greater accessibility to student support services.
Wellbery et al.	2017	Medical Students' Empathy for Vulnerable Groups: Results From a Survey and Reflective Writing Assignment	It was important to know if and how emotional and cognitive aspects of empathy extend to the community, and how these aspects of empathy might enable moral action.	198 Year 1 medical students participated in a two-semester course on social determinants of health. 130 of them completed a pre-test and post-test Social Empathy Index survey. Students' essays were coded and analysed.	1) There was only a significant increase in the contextual understanding of systemic barriers domain (out of the 2 social empathy domains)<br>2) There was an increasing trend of cumulative social empathy scores, though not statistically significant.<br>Thematic analysis:<br>(1) Individual empathy can lay the foundation for empathy for vulnerable groups;<br>(2) civic or moral obligations contribute to social empathy by transcending personal comforts and preferences; and<br>(3) institutional practices that prevent the cultivation of social empathy include curricular, professional, and institutional systems	The short course had limited impact on nurturing social empathy.<br>Empathy must be nurtured at a micro and macro level as students have noted the systematic barriers.
Wellbery et al.	2019	Medical students' individual and social empathy: A follow-up study	Empathy is not only important in the doctor-patient relationship, but also in society. There has been a frequently noted but also contested decline in individual empathy throughout medical education. This study hopes to document changes in	Medical students enrolled in a two-semester course on social determinants of health with a broad focus on health disparities and health policy. Students completed the Social Empathy Index (SEI) pre-test, post-test and 3 years later. The data of 63	The mean "Affective Metalizing" score increased significantly over three years.<br>"Emotional Regulation" (individual empathy) had significantly declined over the same time period.<br>Overall composite social empathy scores increased	The authors speculate that medical students struggle to process the new experiences through medical school and have few resources to cope with these emotionally draining experiences, hence the lag behind in their ability to cope, possibly explaining the significant decrease of

			social empathy, especially individual empathy, after a course on social determinants of health.	students who completed all the 3 surveys was analysed.	significantly over time. "Contextual Understanding of Systemic Barriers" increased significantly in mean score.	emotional regulation in their study. The continued increase of contextual understanding suggests that they are seeing in reality during clinical years the societal problems they had learned theoretically in their preclinical years, thus supporting the need for educational, clinical and community-based interventions to help them stay engaged to the needs of vulnerable groups. The lack of agency of medical students might have resulted in the failure of development of perspective-taking.
Wiecha & Markuns	2008	Promoting medical humanism: design and evaluation of an online curriculum	The loss of empathy in medical students is well documented. Small group discussions have been shown to be effective at preventing this.	Students took part in an online curriculum developed by the authors to promote medical humanism. A control group that took part in the traditional face-to-face curriculum without reflective writing like in the intervention group was set up. Both groups completed surveys before and after the curriculum measuring their self-assessed competence in the 3 domains of humanistic practice the authors were interested in.	1) Students in the online group showed a greater increase in their self-reported ability in all 3 domains of humanistic practice. 2) Most of the students agreed that the online curriculum was easy to use	This study shows that student's competence in key clinical skills related to humanistic practice can be improved with an online curriculum.

Wilkes et al.	2002	Towards more empathic medical students: a medical student hospitalization experience	The development of medical knowledge and increased reliance on technology in the medical field has led to a shift in training away from patient-centredness. Evidence suggests that physicians who experience hospitalization can develop a change in the doctor-patient relationship.	9 second year medical students were admitted on 3 consecutive Saturday mornings with discharge planned for the following Sunday 24-30h later. Only the Director of Hospital Admissions, the Director of<br><br>Nursing, and the attending physician knew that the students were not real patients. A qualitative of the students' hospitalization experience was then collected after their discharge	1) Students were satisfied with the cleanliness of the wards but 7 reported discomfort at sharing a room with a stranger.<br>2) Students reported discomfort at the uncertainty in the process of hospitalization.<br>3) Students reported that the nursing staff were caring, attentive, and professional<br>4) Students felt awkward at the lack of privacy during the hospitalization<br>5) Students reported a concern with improving human aspects of the patient experience	Hospitalisation does improve medical student understanding of patient perspectives
Williams et al.	2014	Empathy levels among first year Malaysian medical students: an observational study	The literature indicates that medical practitioners experience declining empathy levels in clinical practice. This highlights the need to educate medical students about empathy as an attribute early in the academic curriculum.	122 first year medical students took part in a 2h interactive empathy workshop aimed at promoting awareness of empathy. A before and after repeated measures design was adopted to measure outcomes.	The mean JSPE-S scores increased significantly after the workshop	The authors suggest that the cultural differences between their predominantly Asian population and student population in other countries may have contributed to the different baseline empathy levels.
Winefield & Chur-Hansen	2000	Evaluating the outcome of communication skill teaching for entry-level medical students: does knowledge of empathy increase?	Medical communication skills are integral to medical education curriculum. Empathy is essential in doctor-patient communication skills and linked to positive outcomes of helping relationships. The authors describe their efforts to teach empathy to entry-	107 Year 1 medical students participated in a communication skills training programme involving standardized patients (SP) over two 1.5-hour weekly workshops.<br>Students were administered a 10-item	The total empathy score over 10 items improved from 9.97 to 14.44. Baseline skill level did not predict improvement and women scored somewhat higher than men.	Nearly 1/3 of the participants did not improve in their basic empathic response, which may be due to a ceiling effect, or cultural and language barriers between the students and the workshops tutors. The training could have been extended longer to provide more peer modelling, practice

			level medical students and report their outcomes.	empathy test modified from Danish and Hauer pre-test and post-test. Their responses were coded by two trained raters on a scale of 0 to 4.		and feedback to address this. Further work should investigate a better assessment to and investigating the relationship between the responses and actual performance. It is also important to identify the factors affecting students' improvement in skill after training to target those who failed to acquire skill.
Winkel et al.	2016	Narrative Medicine Workshops for Obstetrics and Gynaecology Residents and Association With Burnout Measures	Physician burnout can have a detrimental effect on both the doctor and the patient. Studies have shown that self-awareness is linked to resilience and interventions targeted at self-reflection can lead to reduced burnout and increased empathy. Narrative medicine has been shown to be effective at this.	66 O&G residents participated in the Narrative Medicine curriculum but only 44 had complete data for analysis. A baseline assessment consisting of MBI and a modified IRI was sent to the participants before the intervention, which involved modalities such as sharing of poems and reflective writing exercises. The assessment was repeated 1 year after the baseline assessment.	1) A small increase in empathic concern and a small decrease in perspective taking was observed.<br>2) Participants with high attendance showed a significant decrease in burnout in the domain of Emotional Exhaustion<br>3) Those with a self-care index score higher than the median score showed worse trends in the burnout domain of personal accomplishment.	The results support earlier findings that empathy in doctors decrease over time. However, Narrative Medicine workshops can be effective in curbing burnout and the decrease in empathy. Furthermore, the use of peer discussion groups fosters a culture of support among residents.
Wündrich et al.	2017	Empathy training in medical students - a randomized controlled trial	In medicine, empathic behaviour of doctors has increased patient satisfaction, might lead to better therapeutic alliances and better outcomes. Empathy could also prevent physician burnout. This study aimed to investigated	This is a randomized controlled trial involving 158 year 3 medical students. Students passed through 4 different stations with simulated psychiatric patients (SP). Outcomes were measured by assessment by the SP	1) SP and expert assessment: intervention group was significantly more empathic than the control group on all main outcomes. Gender, age and self-rating of own competence in psychiatry and interviewing skills had	Empathic skills of medical students could be improved through an empathy-focused seminar and empathy skills training with SP.<br>The use of SP was felt to be very authentic, and despite the short training time, the significant effects were found

			if empathy can be taught to medical students in psychiatry and psychotherapy via an empathy skills training with SPs	and experts, and students rated their own empathy level using JSPE-S.	no significant effect<br>2) JSPE-S: no change in the self-assessment of empathy as a whole	to be a high effect size.<br>The lack of change in self-assessment of empathy might be explained by a high self-rated empathy scores at baseline. Self-rated empathy might be less susceptible to change than behavioural empathy skills, which was the focus of this intervention.
Yamada et al.	2018	Changes in Physicians' Intrapersonal Empathy After a Communication Skills Training in Japan	Physician empathy is an essential component of successful patient–physician communication. Previous studies have suggested that educational interventions, like communication skills training (CST), may be effective in maintaining and enhancing physicians' empathy. In Japan, the Cancer Control Act has been law since April 2007; it points out the importance of patient–physician communication. As required by this law, 132 CST workshops were conducted throughout Japan under the authorization of the Ministry of Health, Labour, and Welfare with the goal of improving oncologists' communication skills and decreasing the distress of cancer<br>patients.	507 oncologists participated in a 9h communication skills training, with 383 complete responses received.<br>This is a pretest-posttest quantitative study utilising the Jefferson Scale of Physician Empathy and the Interpersonal Reactivity Index	1) The total JSPE scores and JSPE subscale scores at post-intervention and 3 months after the intervention were significantly higher than those at pre-intervention.<br>2) No significant differences in the JSPE subscale scores at post-intervention and 3 months after the intervention were evident, whereas the total JSPE scores at 3 months after the intervention were significantly lower than those at post-intervention.<br>3) IRI-PT and IRI-EC subscale scores increased significantly from pre-intervention to 3 months after the intervention, whereas IRI-FS and IRI-PD subscale scores showed no significant changes.	Physicians' intrapersonal empathy increased after a communication skills workshop, even at a 3 month follow up.

Yang & Yang	2013	A study of the effect of a visual arts-based program on the scores of Jefferson Scale for Physician Empathy	A literature review demonstrated that there was a need for further studies to evaluate art-based courses in terms of their effects in medical education	110 clerks and interns participated in a 4h visual arts-based intervention involving paintings, stories and movie clips. This is a pretest-posttest quantitative study utilising the Jefferson Scale of Physician Empathy	No significant difference was observed between pretest-posttest JSPE.	Although the results failed to show a positive effect on the JSPE scores, extending the time of the program and increasing the frequency of intervention may be worth studying as most other studies quantitatively investigating the effect of humanities on empathy were in a format of weekly or longer in months.
Yazdi et al.	2019	The influence of role-modelling on clinical empathy of medical interns: A qualitative study	Empathy is an essential component of patient care with benefits for both the patient and physician. Studies have shown a decline in empathy throughout training, yet medical training largely ignores this. Role modelling has been shown to be effective at improving clinical competencies.	Medical interns and clinical professors were invited for semi-structured interviews to find out about their experience with role modelling and clinical empathy.	Students reported that they generally looked for role models with an inspiring personality and attempted to replicate their communication behaviours. Students also noted that negative role modelling occurred especially when they have negative experiences with authority figures.	Role models can have a major impact on student empathy. Students tend to replicate the behaviours of charismatic role models but they will analyse other clinical professors before deciding to replicate their behaviour. Thus, learning from role models has a conscious and unconscious component.
Yu et al.	2019	The Educational Effects of a Pregnancy Simulation in Medical/Nursing Students and Professionals	A competitive work environment in Korea has contributed to a lack of positive attitudes and courtesy towards people who require special considerations, such as pregnant woman. The work culture in Korean hospitals is similar too. Empathy is a necessary trait for physicians and simulation has been shown to be effective than history-based education.	189 medical and nursing students and professionals participated in a pregnancy experience programme which simulates the physical changes of a pregnant woman during the last trimester, completing everyday activities while wearing the pregnancy simulators.<br>This was a quantitative pretest-posttest study using the JSPE-S and	1) The mean JSPE score increased significantly, and there was a significantly higher change in medical students/doctors than nursing students/nurses.<br>2) There was a significant increase in the Physical Difficulty Score and Positive Attitudes Scores<br>3) Professionals were more aware of the physiological changes of pregnancy, subjectively higher empathy and better understanding	This simulation intervention is effective in improving empathy, understanding and awareness of pregnant women among healthcare professional, regardless of gender. Other sources corroborate the effectiveness of simulation to promote empathy and hence it can be a useful learning tool.

				JSPE-HP and 2 surveys developed by the author to measure their perceptions (Physical Difficulty Score), attitudes towards pregnancy and pregnant women (Positive Attitude Score). The participants were also self-assessed on their awareness, subjective empathy and understanding of empathy using a self-developed survey (Perceived Effectiveness Score) after the intervention.	towards pregnant women than students<br>4) 'Major' was found to be a significant predictor of a greater change in empathy level after prep (medicine > nursing) after multiple linear regression analysis.	
Yun et al.	2018	Changing characteristics of the empathic communication network after empathy-enhancement program for medical students	Physicians' empathy-based communication should foster patients' trust in and satisfaction with their doctors, increase treatment adherence, and improve diagnostic accuracy, leading to a more successful treatment response. During medical school and/or residency training, physicians' empathic capacity can be enhanced, preserved, or even eroded. Recent meta-analyses have shown that empathy training can be successful. However, few studies have used the network-based approach to examine training effects for	40 medical students participated in 5 consecutive weekly sessions of an empathy enhancement program (consisting of discussion and roleplay).<br>This is a pretest-posttest quantitative study utilising the Depression Anxiety Stress Scale-21, the Empathy Quotient-Short Form, the Jefferson Scale of Empathy, and the Emotional Expressiveness Scale.	1) Central components of empathic communications were agitation, self-efficacy for predicting others' feelings, emotional concealment, active emotional expression, and emotional leakage.<br>2) DASS-21: depression, DASS-21: anxiety, DASS-21: stress, EQ-short, JSE-S and EES did not show statistically significant changes after the program.	The training effect of the programme is shown by the reduced local segregation and enhanced integration of 'intentional emotional expressivity' with the five hub identified suggests that the program could enable medical students to integrate physicians' emotional expression as an essential component of empathic communication.

			the conceptual integration of multi-dimensional components that comprise empathic communication.			
Zazulak et al.	2017	The art of medicine: arts-based training in observation and mindfulness for fostering the empathic response in medical residents	Empathy is an essential component of patient care with benefits for both the patient and physician. Studies have shown a decline in empathy throughout training, yet medical training largely ignores this. Recent studies have shown increasing interest in using arts-based exercises to improve empathy.	15 residents took part in took part in an arts-based intervention consisting of four 3h workshops held over 1 month. 20 residents attended four 3h didactic academic educational sessions as the control group. Both groups completed the IRI, Compassion scale, and the Mindfulness scale on the first and last days of the programme. A focus group interview was also conducted for qualitative analysis.	IRI and compassion scale showed no significant changes in both groups. The intervention group had lower scores on the mindfulness scale initially but improved after the curriculum.<br>Focus group discussions revealed:<br>1) The intervention provided them with skills to handle stress better<br>2) Participants were better able to understand their patients fully<br>3) Participant hoped to include more specialties under this intervention	Attempts at validating quantitative measures of empathy have yielded mixed results and it is important to include qualitative analysis.
